# Osteology and functional morphology of a transitional pterosaur *Dearc sgiathanach* from the Middle Jurassic (Bathonian) of Scotland

**DOI:** 10.1186/s12862-024-02337-9

**Published:** 2025-01-24

**Authors:** Natalia Jagielska, Michael O’Sullivan, Ian B. Butler, Thomas J. Challands, Gregory F. Funston, Dugald Ross, Amelia Penny, Stephen L. Brusatte

**Affiliations:** 1 School of GeoSciences, University of Edinburgh, Edinburgh, Scotland; 2 Lyme Regis Philpot Museum, Lyme Regis, Dorset, England; 3 Independent Researcher, Limerick, Ireland; 4 Department of Earth and Planetary Sciences, University of California, Davis, Davis, CA USA; 5 Staffin Museum, Ellishadder, Staffin UK

**Keywords:** Flight, Evolution, Jurassic, Pterosaur, Palaeontology

## Abstract

Pterosaurs were the first vertebrates to evolve active flight. The lack of many well-preserved pterosaur fossils limits our understanding of the functional anatomy and behavior of these flight pioneers, particularly from their early history (Triassic to Middle Jurassic). Here we describe in detail the osteology of an exceptionally preserved Middle Jurassic pterosaur, the holotype of *Dearc sgiathanach* from the Isle of Skye, Scotland. We identify new autapomorphies of the flight apparatus (humerus and sternum), which further support the distinctiveness of *Dearc* compared with other early-diverging pterosaurs and describe features, such as the vertebral morphology, shared with later-diverging pterosaurs that probably developed convergently to support a large body size or as a sign of modular evolution. We used extant phylogenetic bracketing to infer the principal cranial and antebrachial musculature, indicating that *Dearc* had large and anteriorly placed palatal musculature that compensated for weak temporal jaw adductors and wing musculature suggestive of flight style reliant on powerful adduction and protraction of the humerus. Comparisons with other pterosaurs revealed that non-pterodactyloids such as *Dearc*, despite their overall conservative bauplans, adapted various flight and feeding styles. The osteology and myology of *Dearc* are indicative of a large predator that flew and hunted above lagoons and nearshore environments of the Middle Jurassic.

## Introduction

The functional anatomy of pterosaurs, volant reptiles from the Mesozoic and the first vertebrates to evolve powered flight, remains poorly understood. This is largely due to a lack of extant animals to act as analogous proxies and a limited number of well-preserved, articulated, three-dimensional fossils permitting functional analysis, such as the mapping of muscle attachments. Exceptionally preserved specimens such as the holotype of *Dearc sgiathanach* (NMS G.2021.6.1—4) from the Middle Jurassic of the Isle of Skye, Scotland, provide a trove of information in this regard. The specimen was named and briefly described in Jagielska et al. [1], which focused on providing a diagnosis of the species and an estimation of its wingspan and growth stage. Here, we build upon the initial description and describe the osteology of the *Dearc* holotype in detail and then use extant phylogenetic bracketing to infer its cranial and antebrachial musculature. This allows us to make comparisons with other pterosaurs and explore the diversity of feeding and flight behaviors in the early-diverging pterosaurs of the Triassic to Late Jurassic.

The holotype specimen of *Dearc sgiathanach* (NMS G.2021.6.1—4) is a well-preserved, articulated skeleton excavated from a bioclastic limestone deposited in a nearshore margin-marine environment [1]. The skeleton originates from the Upper Lonfearn Member of the Lealt Shale Formation, which is part of the Great Estuarine Group. The discovery was made at a site referred to as Brothers’ Point 3 (BP3) in the literature [2] (57.5863°N, 6.1494°W). The animal represents one of the largest Jurassic pterosaurs known from a well-preserved specimen, with a substantial estimated wingspan (> 2.5 m) [1], despite osteological immaturity. A phylogenetic analysis (performed in Jagielska et al. 2022 [1]) nested NMS G.2021.6 within the array of non-monofenestratans commonly called Rhamphorhynchinae, where it falls into a smaller clade of Angustinaripterini [1, 3]. Angustinaripterini are sizeable non-pterodactyloid pterosaurs with low, elongate skulls. Its position within the clade was supported by shared cranial similarities with the Middle Jurassic Chinese *Angustinaripterus longicephalus* [4] and younger *Sericipterus wucaiwanensis* [3]. Since the initial description of *Dearc* [1], new specimens have been discovered challenging the initial phylogenetic placement, emphasizing the importance of the Scottish genus. A study by Hone et al. [5] has shown that *Dearc* fits into the pterodactyloid modular evolutionary continuum, aided by discovery of the first sizeable European darwinopteran *Skiphosoura bavarica* (LF 4157). Phylogenetic revision including *Skiphosoura* recovered *Dearc* closer to monofenestratans and branching after the Rhamphorhynchini. Hone et al. [5] recovered *Dearc* in the clade “Pterodactylomorpha” on account of antorbital shape, total cervical length and the cranial elongation. In this study we will continue to refer to *Dearc* as member of clade Angustinaripterini, retaining its initial phylogenetic placement [1].

The Scottish pterosaur record expanded since the discovery of *Dearc sgiathanach,* with the description of a new darwinopteran, *Ceoptera evansae* [6], from the overlying Kilmaluag Formation of Skye. More non-pterodactyloid pterosaur material was also excavated from the Lealt Shale Formation, of specimen suggesting size even larger than the *Dearc* holotype [7]. These advancements prompt renewed attention of the anatomy, paleobiology, and evolutionary relationships of *Dearc*.

## Materials & methods

### μCT scan

The skull and anterior cervical vertebrae (NMS G.2021.6.2) were separated from the main skeletal block during preparation and subjected to high-resolution X-ray microtomography in a custom-built X-ray μCT scanner at the School of GeoSciences, University of Edinburgh, by IB (elaborated on in [1]). The resulting slices were segmented manually in Mimics (Materialize N.V. 2014). The data show good contrast in regions where the ratio of matrix to bone is low (rostrum), however, the contrast is reduced in the posterior part of the skull (jugal, postorbital, squamosal) where manual segmentation proved to be challenging. Although the posterior skull has reduced contrast, the internal spaces of the brain and ear region are well defined, thus enabling segmentation of brain and endosseous ear labyrinth endocasts and other vacuities. The endocast features were mapped following inferences made by Witmer et al. [8].

### Photography

NMS G.2021.6 was photographed by GF using a Nikon D850 camera with Nikkor 14—24 mm and MicroNikkor 60 mm lenses. The images were created via the automated focus-stacking mode of the Nikon D850 to create enhanced-focus images in HeliconFocus v 7.5.5 of dentition and ungual bones.

### Extant phylogenetic bracket

The musculature placement for the crania and antebrachium in this study was inferred on the basis of the Extant Phylogenetic Bracket (EPB) [9], which encompasses extant ( [10–13] among others) and extinct (i.e., for cranium [14, 15] and antebrachium [16, 17]) Archosauriforms. The osteological correlates refer to texture changes, ridges, depressions, and other topographic landmarks present on the bone.

### Ungual curvature

The measurements of ungual curvature were aided by the program DinoLino developed by Cobb & Sellers [18] for measuring inner (ventral) and outer (dorsal) ungual curvatures on the two-dimensional plane. The inner curvature is measured between the base of the flexor tubercle and the tip of the ungual bone, with an intersection of the bisector of the curved surface. This is on the basis of Feduccia’s modified method [19]. Pike and Maitland’s method [20] was used to quantify the outer ungual curvature, starting from the proximal termination of the dorsal ungual bone stretching to the ungual tip and with the bisector between the two points. The values for *Dearc* were compared to those for species and behavioral groups of extant animals (as in Cobb & Sellers [18]).

## Results

### Systematic paleontology

Pterosauria Owen, 1842 [21]

Breviquartossa Unwin, 2003 [22]

Angustinaripterini He, 1983 [4]

### Type species

*Dearc sgiathanach* Jagielska et al. 2022 [1]

### Type material

**Fig. 1:**
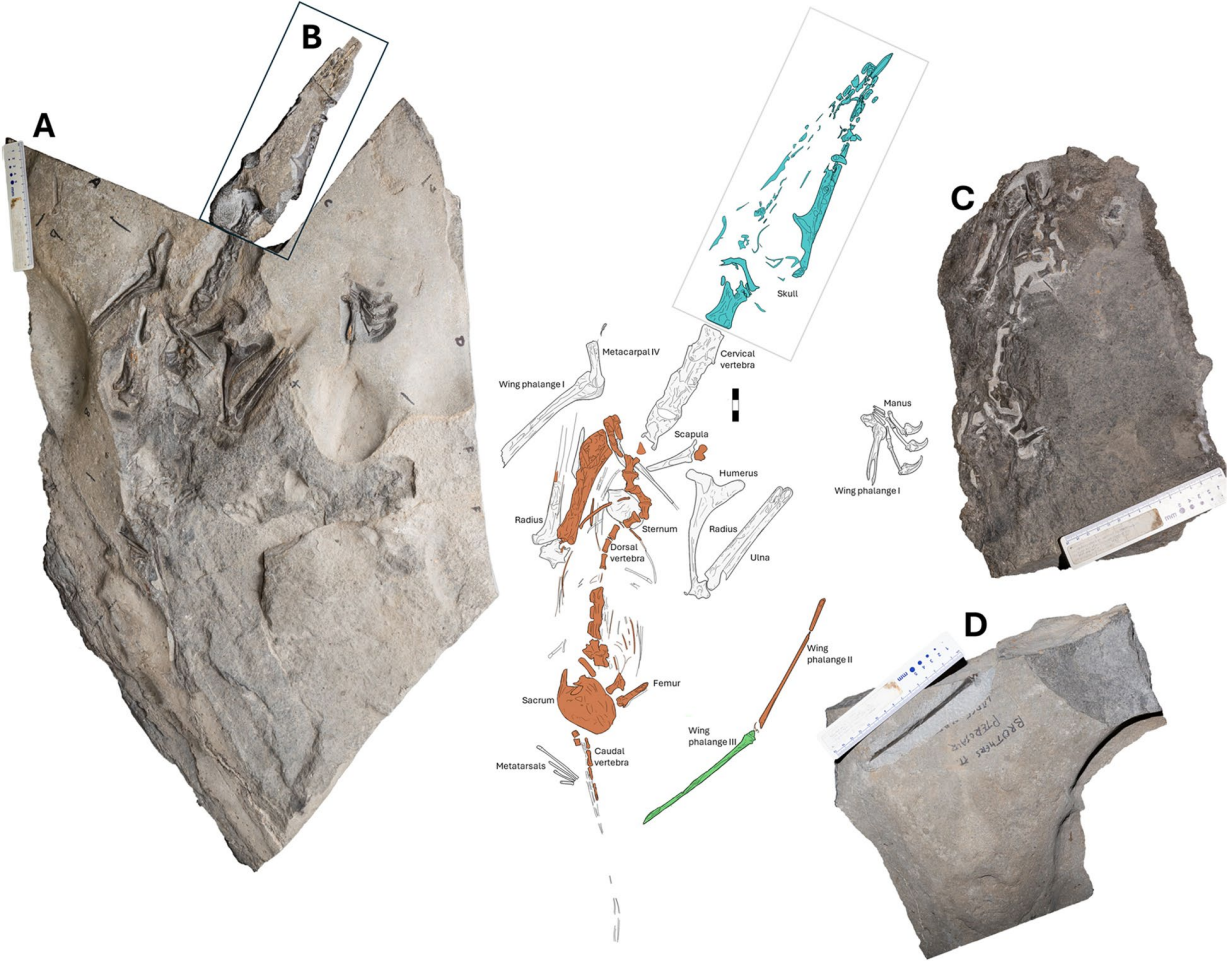
A photograph and corresponding illustration of NMS G.2021.6. **A** a photograph of NMS G.2021.6.1 or “the main slab” showcasing the postcranial material in the dorsal view, presented in white in the illustration; **B** a photograph of NMS G.2021.6.2, a three-dimensional skull with the anterior-most cervical vertebra, highlighted in blue in the illustration; **C** a photograph of NMS G.2021.6.3 or “the counterslab” of the postcranial material in ventral orientation, highlighted in orange in the illustration; **D** a photograph of NMS G.2021.6.4 showcasing a singular third wing phalange in the lateral view, highlighted in green in the illustration. Scale bar 30 mm

NMS G.2021.6.1—4 (Fig. 1) is an associated skeleton preserved in three dimensions, with NMS G.2021.6.1 referring to the postcranial material (Fig. 1A), NMS G.2021.6.2 to the skull (Fig. 1B), NMS G.2021.6.3 to the postcranial counterslab (Fig. 1C) and NMS G.2021.6.4 to a singular phalange (Fig. 1D). The skeleton is encased in a hard limestone, and many elements are visible in limited views. The skeleton is almost entirely in articulation, including the skull (which retains delicate palatal, ceratobranchial and neurocranial elements), a complete cervical vertebral series, well-preserved elements of paired forelimbs with a complete right manus and partially preserved wing phalanges, a disarticulated dorsal vertebral series and ribcage, and a poorly preserved sacral, pelvic, tail and hindlimb region.

### Revised diagnosis

The clade-wide”Angustinaripterini” traits shared between *Angustinaripterus longicephalus* [4]*, Sericipterus wucaiwanensis* [3] and *Dearc sgiathanach * [1] were noted in Jagielska et al. 2022 [1] and include a low and elongate skull (height‒length ratio < 0.2); large antorbital fenestra (20‒35% skull length and > 80% orbit dorsoventral height); lacrimal process of the jugal nearly perpendicularly inclined (90°–110°) to the jugal body; strongly inclined quadrate (130°–140° relative to the maxillary long axis); cervical vertebrae with considerable changes in the length‒to-width ratio across the neck (1.8 to 1.2, from anterior to posterior); and humeral diaphysis slender with a muscle scar tubercle. The species-specific autapomorphies include tritubular vomers with “trident-shaped” precapillary contact, a pre-choana depression on the palatal surface of the maxilla, enlarged optic lobes expanded anteroposteriorly, and the fourth metatarsal being more robust (diameter 2.5 ×) than the metatarsals one to three. Through our detailed look at the anatomy and osteology we can add two new autapomorphies, such as the deltopectoral crest being proximally expansive and taller than the ulnar crest, and the sternum bearing exhibiting an anteriorly deflected frontal margin. In the phylogenetic assessment by Hone et al. [5] *Sericipterus wucaiwanensis* shifted to Rhamphorhynchini, while *Angustinaripterus longicephalus* and *Dearc sgiathanach* were relegated an “unknown clade” encompassing Monofenestrata. Due to humeral and cervical characters shared by *Dearc* and *Sericipterus*, in this paper, we will continue referral to the Angustinaripterini clade.

### Cranium

**Fig. 2:**
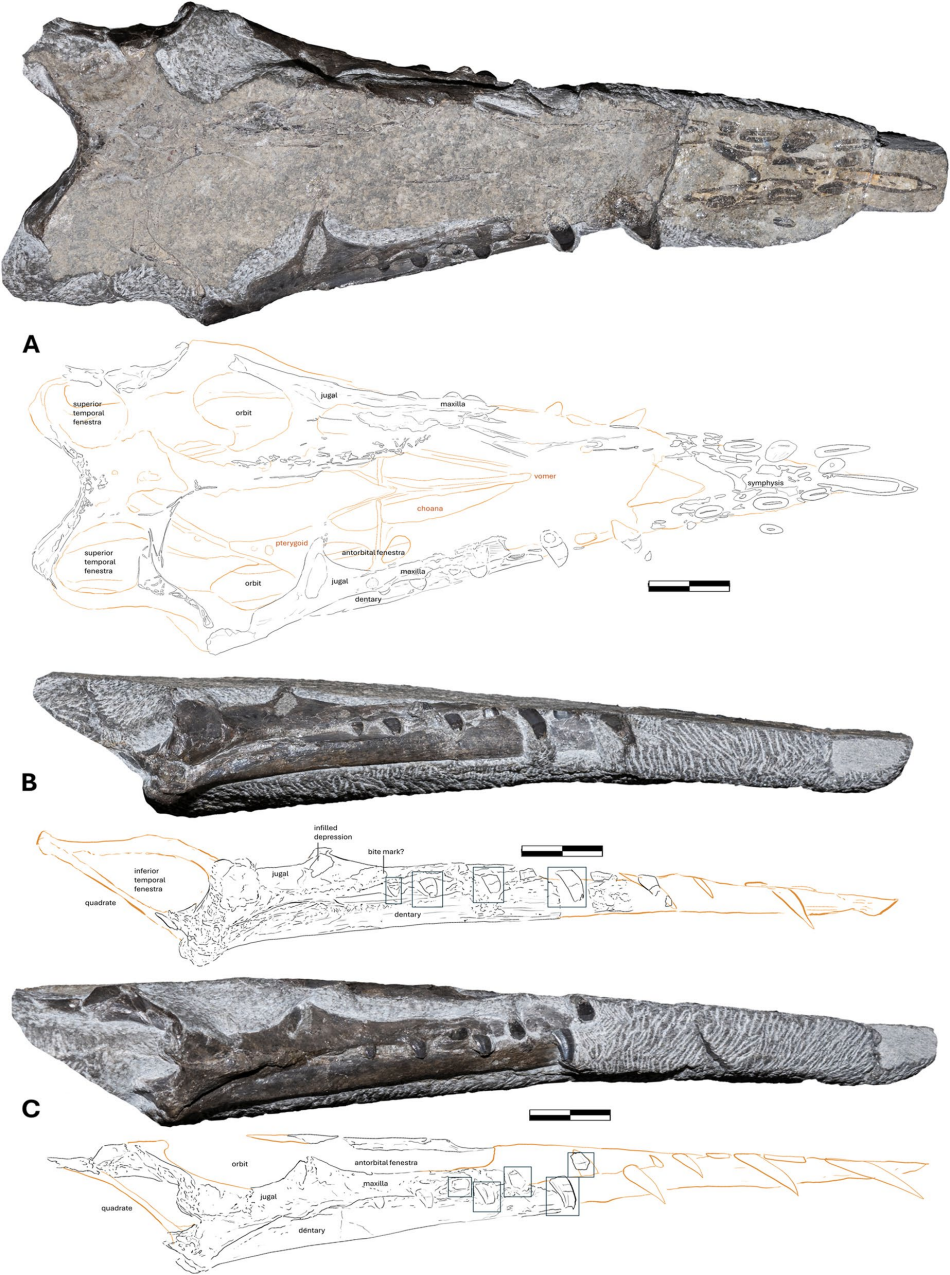
Photographs and illustrations of the skull and associated elements (NMS G.2021.6.2). **A** NMS G.2021.6.2 as seen in the dorsal view; **B** right lateral view; **C** left lateral view. Scale bar 20 mm

The skull (present on a separated slab, NMS G.2021.6.2 (Fig. 1B)) is well preserved and not severely deformed (Fig. 2A-C). It is largely complete, missing only the anterior dorsal surface, which was worn away by tidal exposure. The dorsal and lateral orbital areas, along with the ventral mandible articulation, were prepared and can be readily assessed visually.

The skull is 222 mm long anteroposteriorly [1], as measured on the basis of the fully preserved lower jaw, from its anterior tip to the termination of the posterior-most extension of the parietal bones of the cranium. This would rank it among the longest non-pterodactyloid skulls on record, being comparable with the nearly equally sizeable *Angustinaripterus longicephalus* (est. 209 mm) [4]; *Dimorphodon* NHM PV OR 41212 (220 mm) [23] and the largest recorded *Rhamphorhynchus* NHM PV OR 37002 (195 mm) along with estimated lengths derived from partial material (*Harpactognathus* (280–300 mm) [24] and *Sericipterus* (210 mm) [3]. The skull is 35 mm tall dorsoventrally at its maximum, and although this would be a slight underestimate of genuine depth owing to the loss of part of the dorsal surface of the skull, it is clear that the skull has a low and elongated profile. Proportionally, the skull length is close to the length of the preserved dorsal vertebra (235 mm). The low skull profile is also comparable to that of *Angustinaripterus*, suggesting a clade-wide trait. The skull width varies from 60 mm posteriorly to 50 mm medially and 10 mm anteriorly, forming a triangular shape in dorsal view.

The maxilla of NMS G.2021.6.2 is straight and has a constant dorsoventral depth of 15 mm. It has a total preserved anteroposterior length of approximately 55 mm, with three alveolar spaces. The antorbital fenestra can be delineated from its ventral margin, measuring approximately 50 mm proximodistally. The fenestra’s ventral margins are elongate and flat, running parallel to across the skull. It is distally bordered by the lacrimal process of the jugal. It bears similarities in alveolar spacing and morphology to a specimen classified as Rhamphorhynchidae indet. (OUMNH J.28409) from the Taynton Limestone Formation [25], suggesting a wider geospatial distribution of Jurassic pterosaurs with this type of snout morphology.

The most visibly exposed regions of the skull are the lateral surfaces of the left and right jugals. The jugal is approximately 70 mm long, with 35 mm tall antorbital and 40 mm tall postorbital sections. There is a round depression on the right side of the skull around the jugal–maxilla intersection (Fig. 2B), which is either a taphonomic artifact or a shallow healing bite mark. The jugal postorbital process bows posteriorly from the body and the long axis of the jugal. The lacrimal process is angled approximately 120° laterally to the jugal body, reclining in the opposite direction to the quadrate. The jugal lacrimal process on both sides has a deep recession that has been infilled with matrix, leading to the illusion of a fenestra (Fig. 2B).

The jugal in *Dearc* differs from that of other three-dimensionally preserved pterosaurs, such as *Cacibupteryx* (IGO-V 208). In *Cacibupteryx,* the jugal ventral to the orbit is reduced to a thin bar [26], and the body of the jugal does not lie in line with the maxilla, as in *Dearc*. This also applies to *Rhamphorhynchus*, where the maxilla and the body of the jugal do not lie in the horizontal continuum; instead, the jugal deflects ventrally relative to the maxilla in its posterior section (MTM V 2008.33.1, CM 11434, NHMUK PV OR 37002, WDC CSG 255, among others [27]). The jugal and the associated antorbital fenestra are also different in *Dearc* than in *Rhamphorhynchus*, as the latter has a diminutive antorbital fenestra (as seen in WDC CSG 255; MTM V 2008.33.1, among others) in the shape of a small triangular slit less than a third-to-a-half of the orbit height [28], differing from the inferred elongated, sizeable opening seen in *Dearc.* In *Rhamphorhynchus* (NHM PV OR 37002), the maxilla increases in dorsoventral width anteriorly, whereas the thickness remains constant throughout its length in *Dearc.* The specimens of *Scaphognathus* (SMNS 59395) and *Dorygnathus* (UUPM R 156, SMNS 55886 and others [29]) exhibit jugal conditions that are more similar to those of *Dearc*, with the maxilla and jugal in line with a straight ventral margin. *Scaphognathus* also bears sizeable antorbital fenestra with evenly spaced alveoli.

While the lacrimal in NMS G.2021.6.2 is missing, the remaining margins of the postorbital and jugal delineate a circular orbit. The orbit is approximately 36 mm in diameter. The supratemporal fenestra is circular and 15 mm in diameter. The lateral temporal fenestra becomes narrower dorsally, creating a teardrop-like shape (Fig. 2A-B), with a flat posterior margin. It is over 30 mm long dorsoventrally and 14 mm wide anteroposteriorly at its widest point. The elongate tear-like shape and relative size of the fenestra resemble those of juvenile *Scaphognathus* (SMNS 59395), differing from *Angustinaripterus* (ZDM T8001), where the fenestra narrows in the opposite direction. It is bordered by a quadrate reclining posteriorly by ~ 130°–140° to the ventral margin of the jugal. The quadrate is strongly posteriorly reclined in *Dearc* relative to *Cacibupteryx* (IGO-V 208) and *Parapsicephalus* (GSM 3166), where the bone is almost vertical to the body of the jugal; in other Jurassic species (*Angustinaripterus*, *Dorygnathus, Rhamphorhynchus*), the angle varies from 130–135° becoming increasingly more inclined posteriorly in monofenestratans [30]. The supratemporal temporal fenestra of NMS G.2021.6.2 are rimmed by parietal bones, which fold anteriorly by and are incised anteriorly. Given the poor quality of the posterior cranial section in the scan, detailed descriptions of select braincase bones (exoccipital, opisthotic, pseudomesethmoid and such) will await improved scan quality.

### Palate

**Fig. 3:**
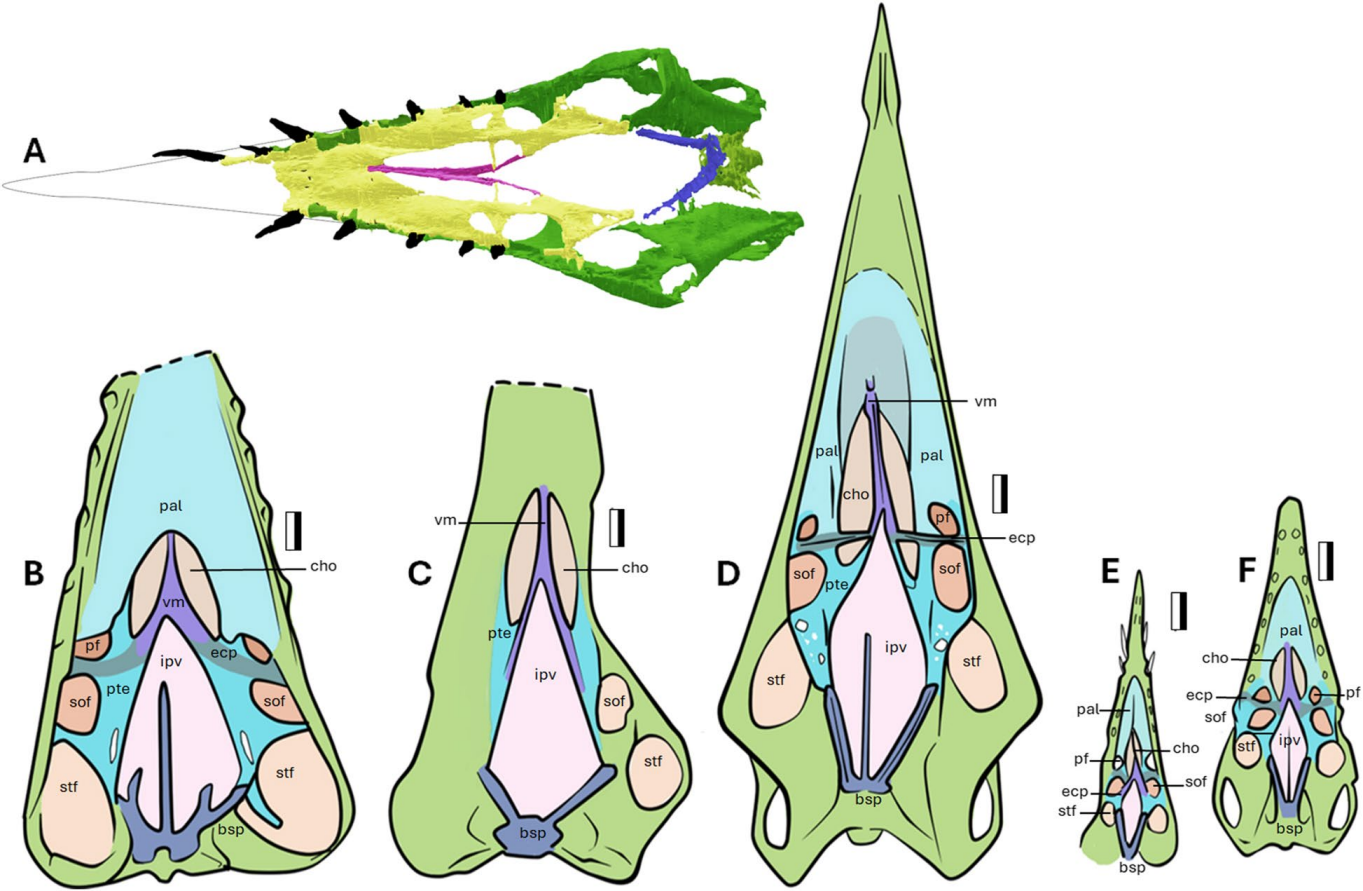
The palatal view of the skull of NMS G.2021.6.2 and comparative assessment of the pterygoid region. The palatal region of NMS G.2021.6.2, **A** as seen in the ventral view. The comparative, to scale, pterosaur palatine and palatal regions in the ventral view. From left to right: **B** *Cacibupteryx* (IGO-V 208); **C** *Parapsicephalus* (GSM 3166); **D** *Dearc* (NMS G.2021.6.2); **E** *Rhamphorhynchus* (NHM R 2786); **F** *Rhamphorhynchus* (CM 11434). Abbreviations: bsp—basipterygoid; cho—choana; ec -, ectopterygoid; ipv—interpterygoid vacuity; pal—palatine; pf—posterior palatine fenestra; pte—pterygoid; stf -subtemporal fenestra; sof – suborbital fenestra; vm—vomer. Scale bar 10 mm

In NMS G.2021.6.2, the palatal region is preserved in situ within the articulated skull and enclosed by a matrix, which protects it from extensive damage or deformation. It is preserved in three dimensions and is the best-preserved palate of a non-pterodactyloid pterosaur known to date (Fig. 3A). Pterosaur palatal regions are frequently found in a flattened and disarticulated state. Currently, our understanding of non-pterodactyloid pterosaur palates is restricted to ventral views of *Parapsicephalus* (GSM 3166, explored in Newton, 1888 [31, 32], *Cacibupteryx* (IGO-V 208 [26]), *Rhamphorhynchus* (NHMUK PV R 2786, CM 11434, Padova, no. 6580 [33]), and others based solely on individual disarticulated pieces (i.e., [23]) or partial composites (e.g., *Doryhgnathus*: SMNS 18969, 50184, 50914, 51827) [34]. Owing to the fragility of the palate of *Dearc* and its placement within the articulated skull, it could not be prepared and had to be manually digitally segmented from μCT scan data (as seen in Fig. 3A).

In *Dearc* the palatines enclose a heart-shaped midline choana cut centrally by a forking vomer (Fig. 3D). Posteriorly, there is a large diamond-shaped midline interpterygoid vacuity bordered by a robust basisphenoid, with the surrounding pterygoids helping to define three fenestrations on each side of the palate (postpalatine, suborbital, subtemporal) (after Ősi et al. [34]). The ectopterygoid is a thin bone stretching from the anterior segment of the pterygoid that contacts the posterior segment of the vomer. On each side of the palate, it acts as a separating bar between the postpalatine fenestra and suborbital fenestra. The ectopterygoid penetrates the choana and meets the vomers at an almost perpendicular (95°) angle (Fig. 3D). The ectopterygoid extension is thin and slender, 27 mm long mediolaterally, and contorts around its axis. The vomer is a thin bone that is concave on its lateral sides, connects the palatines to the pterygoids, and cuts medially through the choana opening. In *Dearc,* it is composed of three cylindrical rods converging at an anterior point in a “trident”-shaped dorsal precapillary contact. This feature has not been observed in other pterosaurs and was considered autapomorphy [1]. The choana is one of the largest openings in the palate and is placed beneath the antorbital fenestra. It is a 45 mm long vacuity between the palatines and pterygoids, divided by the vomer. It is narrow (17 mm at maximum), with an elongated heart shape. The region anterior to the choana causes a lobate topographic depression in the palate, which was also noted but not delineated in *Rhamphorhynchus* CM 11434 (Fig. 3E-F).

There are three major paired fenestrations defined by each pterygoid, increasing in size posteriorly. The postpalatine fenestra measures 6 by 5 mm anteroposteriorly, followed by suborbital fenestra at 16 by 9 mm and a posterior-most subtemporal fenestra at 25 by 9 mm. The pterygoid borders contact the jugal at a perpendicular angle. Posteriorly, the pterygoid is riddled with small fossae, a feature also observed in other pterosaurs (in *Cacibupteryx* labeled as the “postpalatal fenestra” [26])*.* In *Dearc,* the basipterygoid opens at 44°. This angle is narrower than that of pterosaurs such as *Parapsicephalus* or *Cacibupteryx* (74° and 85^o^ respectively) (Fig. 3B-C), twice as wide as that of *Rhamphorhynchus* (20—28°) (Fig. 3E-F) and similar to that of the transitional *Allkaruen koi* (MPEF-PV 3613) with 35° [35]. Although there are no visible suture lines between the jugal, maxilla, quadratojugal and quadrate, there are clear distinctive unfused lines between the quadrate complex, pterygoid and basipterygoid. This articulation is observed in most pterosaurs with well-preserved palatal regions [34].

### Ceratobranchials

**Fig. 4:**
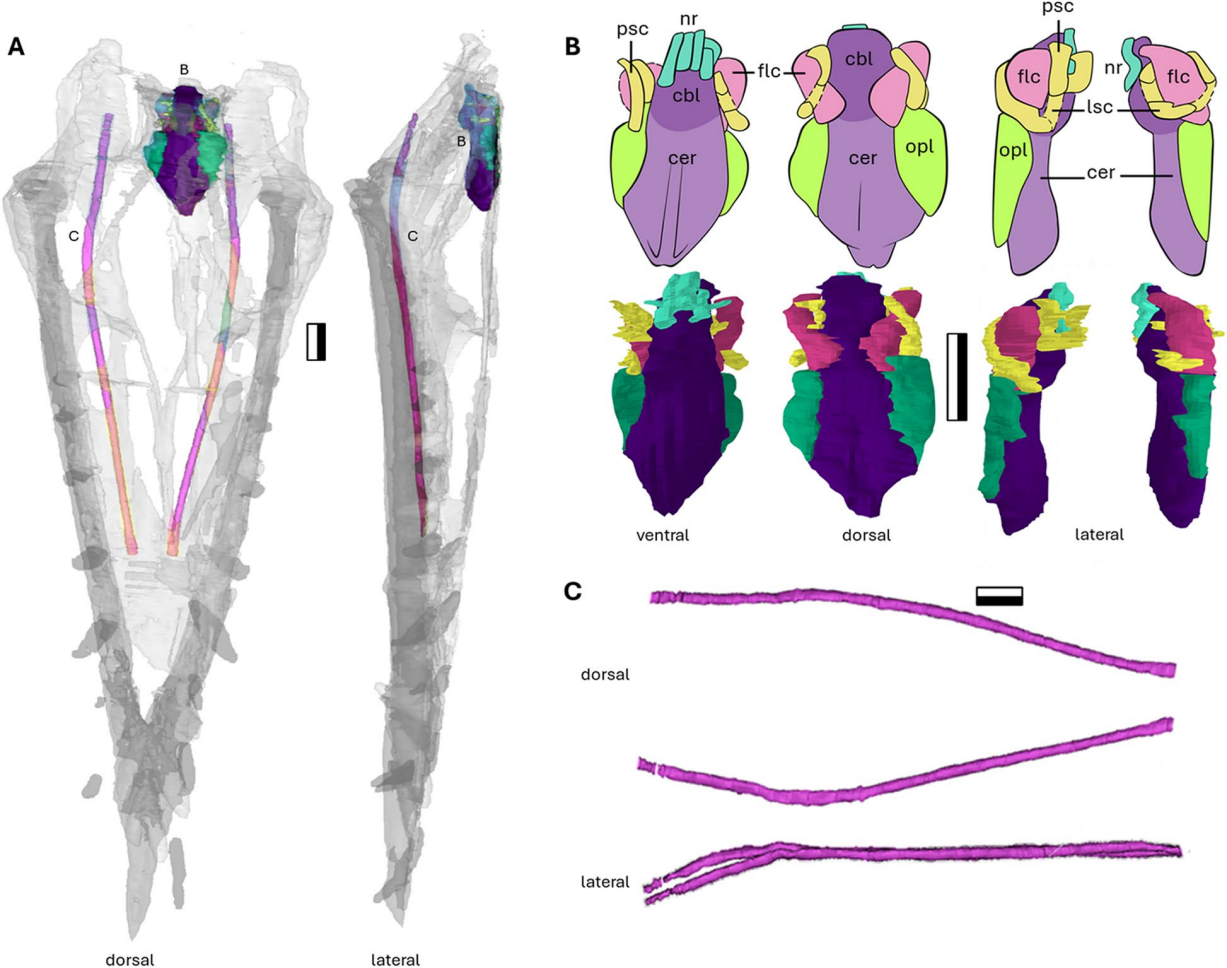
The internal cranial features of NMS G.2021.6.2. The segmented NMS G.2021.6.2 with highlighted endocast (purple) and ceratobranchial bone (pink) seen in, **A** the dorsal and lateral view. The segmented μCT scan of the endocast infilling and semi-circular canals, along with interpretative illustrations, **B** in ventral, dorsal, and lateral orientations. The ceratobranchial bones in, **C** dorsal and lateral orientation. Abbreviations. cbl – cerebellum; cer – cerebrum; lsc – lateral semi-circular canal; nr – cranial nerves; opl – optic lobe; psc – posterior semi-circular canal. Scale bar 10 mm

The delicate paired ceratobranchials lie in the cranial cavity of NMS G 2021.2.6.2 and measure 144 mm in anteroposterior length (Fig. 4A). The ceratobranchials in pterosaurs are morphologically simple, elongate and slender rods (Fig. 4C), and this is also the case in *Dearc*. In NMS G.2021.6.2, the ceratobranchial bones do not bear any scarring or enlarged areas associated with any muscular attachment. The paired bones are circular in cross-section and flatten at the anterior apex. The brachia curve dorsally at 160° posteriorly and bend buccally 160° in dorsal view (Fig. 4C). The brachium is not dissimilar to that of *Dorygnathus* (SMNS 50702) or *Scaphognathus* (SMNS 59395) [36].

### Endocranium

NMS G.2021.6.2 preserves the brain endocast cavity, which we segment from μCT data (Fig. 4A) with the brain regions delineated following inferences made by Witmer & Thomason [8]. The interpretations must be taken with caution, however, as the endocranial cavity in NMS G.2021.6.2 has been truncated dorsally by the recent tidal erosion, and likely experienced at least a minor degree of dorsoventral flattening. The preserved cavity space is approximately 24 mm long anteroposteriorly (Fig. 4B).

In our reconstructed endocast, the cerebellum is a relatively deep and round structure at the back of the brain, from which a flocculus protrudes on each lateral side. The cerebellum is capped by the optic lobes close to the dorsally placed cranial nerve openings. The flocculus makes up a third of the length of the segmented endocast (Fig. 4B), close to being in line with the length of the cerebral body, which is similar to the condition in *Rhamphorhynchus* [8], *Caelestiventus* (BYU 20707) [37] and the lagerpetid outgroup *Dromomeron* (TMM 31100–1334) [38, 39].

The optic lobes of the midbrain originate from the cerebrum‒cerebellum intersection point and extend from the lateral sides of the cerebrum. The lobes are thin and curved, wrapping around a substantial proximodistal length of the cerebrum (Fig. 4B). Among all endocast regions, the optic lobes are the most peculiar, manifesting as flat, curved extremities, being more extensive and morphologically different from the optic lobes observed in other pterosaurs. *Dearc* had relatively large orbits and probably required increased visual neural support. Similar morphology has been noted in other pterosaurs; for example, in the reconstruction of an infilled *Parapsicephalus* endocast also shows an elongated thin extremity in dorsal and lateral views (as illustrated in Hopson, 1979). The *Parapsicephalus* endocast has not been manually segmented and therefore cannot be used as a definitive comparison. In comparison, the optic lobes of *Rhamphorhynchus,* as inferred by Witmer & Thomason [8] and Codorniú [35] show a small triangular feature in line with the cerebrum. The optic lobes in Triassic *Caelestiventus* have also been described to lie in line with the cerebral lobes but remain unflexed ventrally or laterally [37]. In *Allkaruen,* the condition reflects that of Cretaceous pterodactyloids, with optic lobes, which are again smaller and triangular but set below the long axis of the cerebrum [35].

The cerebrum is a set of elongate, shallow paired lobes with a medial separating furrow (Fig. 4B). The full anterior extent of the cerebrum is unknown, given the lack of sufficient preserved bone to delineate its terminus.

There is a set of poorly preserved tubular cavities running underneath the endocast complex (Fig. 4B), which can be interpreted as cranial nerve tracts. These are tangibly visible in the scan slices, but like the semicircular canals, they are not clearly visible and are partially collapsed. The nerves stem from the posterior end of the endocast, ventrally to the cerebellum close to the occipital region. At least three channels can be seen opening posteriorly on the horizontal plane.

The overall morphology of the brain endocast is the same as that observed across the early diverging pterosaur taxa, where the optic lobes are level with the bulk of the forebrain. This would make the *Dearc* endocranium less akin to the more derived *Allkaruen* and closer to the nonvolant lagerpetid, *Dromomeron gregorii* (TMM 31100–1334 [38, 39]), a close relative of pterosaurs. This level-lobe condition is also observed in one of oldest pterosaur clades, dimorphodontids, in the only segmented endocast of a Triassic pterosaur, *Caelestiventus*. *Caelestiventus* stands out, however, for having an elevated cerebellum (a condition we cannot assess in *Dearc*, due to erosion) and anteriorly sloping olfactory bulb [37]. Interestingly, the *Rhamphorhynchus* specimen that has been described [8], despite being less than half the size of *Dearc*, has a remarkably similar endocranial morphology, with similarly sized and distributed cerebral lobes, cerebellums and flocculi (with associated semicircular canals). This finding might suggest that ontogeny and size do not have a substantial effect on general brain morphology in non-pterodactyloids. There have yet to be μCT-derived endocasts constructed for *Cacibupteryx* and *Parapsicephalus*, despite both being preserved in three dimensions, which is surprising given that the infilled endocast of *Parapsicephalus* has been observed throughout the literature [40, 41].

### Inner ear

The inner ear cavities (endosseous labyrinth) have been used to speculate on the sensory, locomotor, stabilizing and auditory capabilities of extinct animals [42]. In all known pterosaurs and their close relatives [38, 39] the semicircular canals are large and arching. The lateral canal is horizontally inclined 80—90° at the anterior–lateral canal intersection (Fig. 4B), as observed across Pterosauria [35]. The long axis of the lateral semicircular canal is flat and parallel to the long axis of the skull. The anterior semicircular canal is the longest of the three principal canals; it incises medially and caps the flocculus. The posterior semicircular canal rims the posterior margin of the flocculus. The canals are slender and no thicker than 1 mm in diameter.

### Dentary

The lower jaw lies in articulation, in a closed-mouth position with the cranium on the NMS G.2021.6.2 block. The jaw rami are straight in lateral view with a slight concavity (174°) at the medial section and a consistent dorsoventral thickness of 10 mm. A single ramus houses approximately 6–7 alveolar pairs, two of which are held in the symphysis. The jaw is extensively pneumatic, as shown in the scan cross sections, and house sets of variably sized cavities in the posterior jaw section. The dorsal and ventral extremities of the dentary bone wall are relatively thick. The rami converge on a keeled symphysis at an angle of 30° falling within a range of values across rhamphorhynchines (pers. obs.), varying from 17° in some *Rhamphorhynchus* (GPIT 1645⁄1, BSPG 1977 I 226) specimens to 37° in *Bellubrunnus* (BSP–1993–XVIII–2). The symphysis in *Dearc* makes up 30% of the entire dentary, a value not dissimilar to that of other non-pterodactyloids. The symphysis has a rough pitted surface that indicates a keratinous sheath in life, which can lead to the creation of a curved, keratinous prow [27, 43]. It might have performed a similar function to the hook on the terminal beaks of piscivorous birds [44] to stabilize slippery prey. The morphology of the symphysis is more similar to slender, narrow condition such as that in *Rhamphorhynchus,* and not deep, broad, and arch-like shapes, as seen observed in numerous scaphognathids [45, 46] or an elongate narrow point in monofenestratans [47]. The posterior section of the jaw forms a globular, condyle-like, rugose area.

### Mandibular neurovascular canals

On the basis of the μCT data, the most delineated canals are present in the thickened parts of the mandible dorsal and ventral to the mandibular rami and are most prominent in the posterior half of the lower jaw. In archosaurs, the canals house mandibular trigeminal nerves and arteries [48–50]. The cavities are circular and bifurcate into two to three channels at the medial point of the jaw. The largest central cavity likely channeled Meckel's cartilage. Only a handful of studies have explored mandibular canals in pterosaurs in detail [49]. Martill et al. [51] also noted the neurovascular foramina on the rostrum and symphysis of the Cretaceous pterosaur *Lonchodraco*, which connected with the trigeminal nerve in the dentary and aided rostral sensation used in foraging [51]. The surface of the edentulous symphysis in *Dearc* cannot be accessed to identify the presence of small sensory foramina. However, a triangular cavity located in the dorsal region of the symphysis could have been involved in an analogous function.

### Dentition

**Fig. 5:**
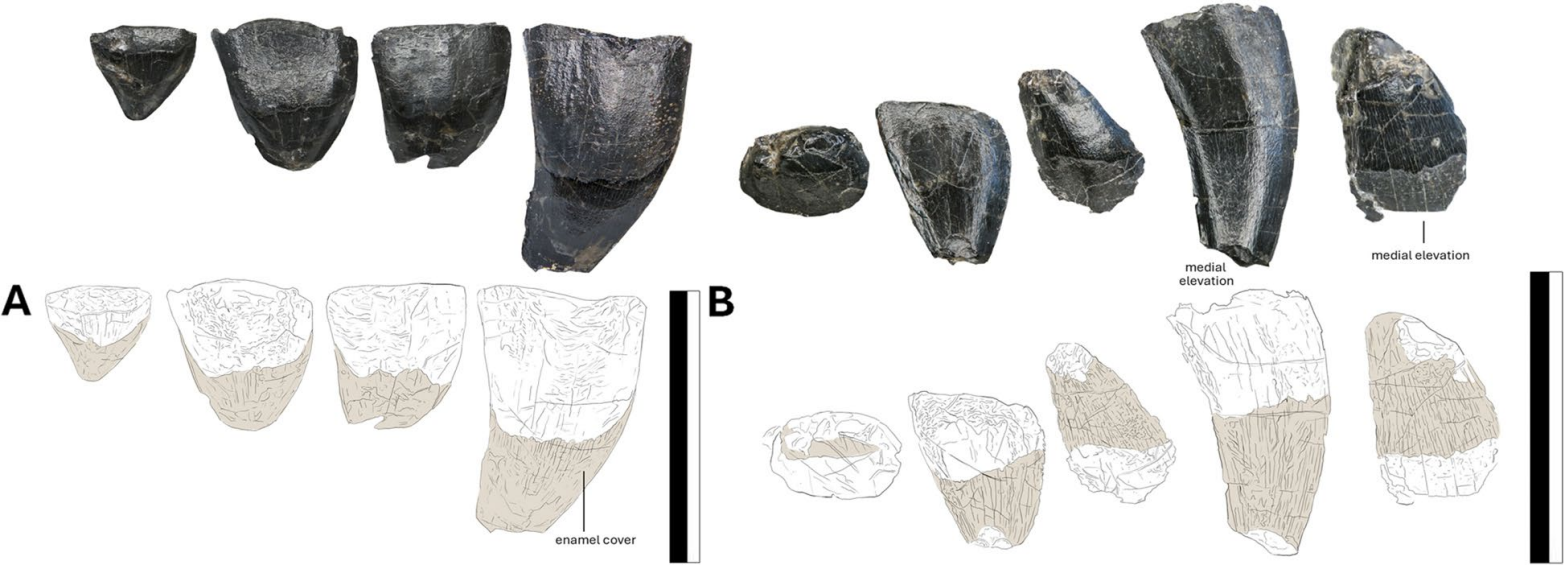
The high resolution photographs of the posterior teeth of NMS G.2021.6.2. The exposed teeth of the dentary and premaxilla, **A** of the right lateral and, **B** the left lateral views. The elements in beige represent the enamel cover. Scale bar 10 mm

There are 6–7 teeth per ramus (with one possible replacement tooth visible internally) (Fig. 2), giving the animal a dental formula of at least 13 teeth in the dentary. The dentition has been severely damaged by erosion: the upper jaw premaxillary dentition were eroded to tooth mid-sections, the lower jaw preserves only the roots of the symphyseal teeth. The spacing between the tooth alveoli is somewhat irregular, fluctuating between the equivalent of two to four alveolar spaces in the maxilla, and the teeth become more packed anteriorly. There are no discernible changes in the tooth morphology between the upper and lower jaws. There are two distinct types of teeth in the upper and lower jaws: premaxillary elongate fangs and prism-shaped maxillary pegs (Fig. 5) and their symmetrical dentary equivalents. The upper jaw dentition, however, extends more posteriorly than the lower dentition by two pairs, covering the entire length of the antorbital fenestra.

The premaxillary teeth and their dentary equivalents are smaller than the posterior set. The premaxillary teeth have smooth buccal faces and taper toward the tip, retaining a slight distal curvature. The teeth differ in dimension, becoming larger and more recurved mesially. The largest preserved tooth measures approximately 19 mm in apicobasal length; it is located in the anterior section of the jaw, but its full size remains unknown. Similar teeth, albeit more sizeable (70 mm), have been recovered from the Callovian Balabansai Svita [52] and Bathonian Stonesfield Slate [25]. *Sericipterus* (IVPP V14725) also had larger teeth (53 mm) [3], and unlike *Dearc*, all teeth retained a strong curvature. The dental condition of large anterior recurving fangs and prism-like posterior teeth in *Dearc* closely resembles that of *Angustinaripterus*, albeit in *Angustinaripterus,* the teeth are more tightly anteriorly placed, unlike some larger specimens of *Dorygnathus* [29]. Similar conditions have also been observed in sizeable (mature) members of *Rhamphorhynchus* (i.e., NHMUK PV OR 37002).

The teeth that stem from the maxilla are small, squat and straight. The posterior-most teeth are equant in size (3.8 by 3.8 mm) (Fig. 5). Most have chipped tooth crowns (Fig. 5). The teeth are symmetrical, with a medial elevation (Fig. 5), and have short, shallow roots. The enamel coverage reaches over 50% (Fig. 5). This cover decreases to one-third of the tooth, restricted to the apices, in the mesial-most teeth. The enamel preserves light longitudinal stripes, which leave no topographic changes. The cover protected the tooth from erosion and degradation, as the elements recovered above the cap are in poorer condition than those below it.

The enamel exhibits signs of cracking and spalling on all teeth, leaving sets of lines down the long axis of the tooth and stepped contact surfaces (Fig. 5). Similar wear patterns, “short and conchoidal, extending to the apex of the tooth” [53], were noted in *Dorygnathus* specimens (SMNS 81840). This, among other inferences, has been interpreted as wear patterns caused by interactions with hard food sources [54, 55]. The dentition and its inferred use observed in *Dearc* are analogous to those of *Dorygnathus* [53] and adult *Rhamphorhynchus* [56], suggesting that *Dearc* was capable of feeding on harder prey items. The differentiation between two tooth morphologies in the maxilla and premaxilla might reflect an overall trend in which non-pterodactyloid teeth become more differentiated with jaw elongation [57]. No sign of antemortem tooth-on-tooth occlusion was observed in *Dearc*, but the teeth likely meshed into a tight interlocking bundle at the tip of the snout, indicating a lack of shearing dentary motion. A cluster of tightly interlocking fangs has been proposed to act as a “fish grab” [40, 53]. The penultimate tooth distally does not have notable wear, suggesting that it might not have been used as extensively in feeding.

### Vertebrae

**Fig. 6:**
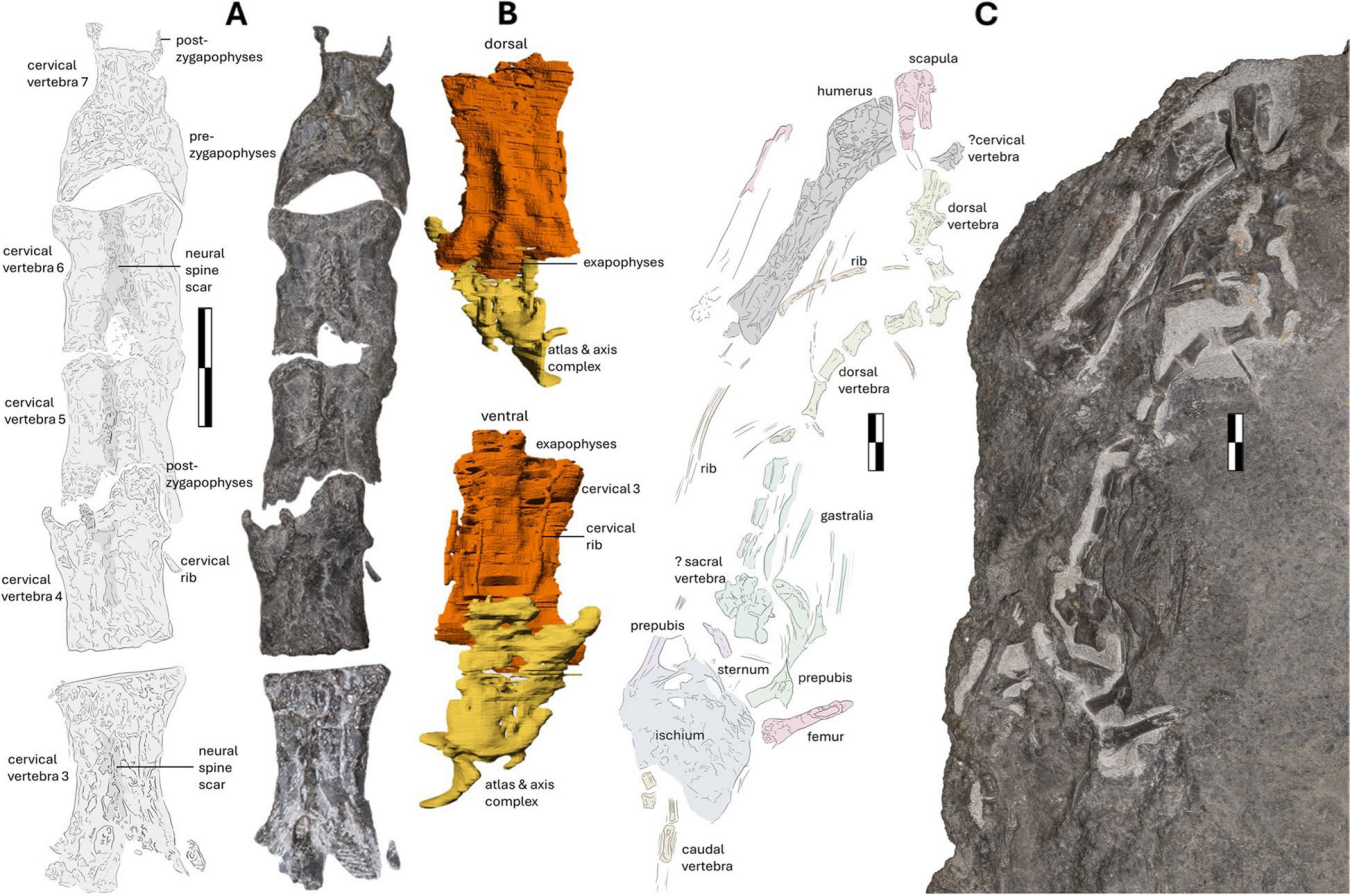
The vertebral series of NMS G.2021.6.1–3. A dorsal view of cervical vertebra (3 to 7) of NMS G.2021.6.1–2, **A** interpretative annotated illustration and a photograph. **B** the atlas-axis (yellow) and the third cervical (orange) as seen after CT segmentation, in the dorsal (top) and ventral (bottom) views. **C**, the ventral counterslab of NMS G.2021.6.3 with interpretative annotated illustration and photograph. Scale bar 10 mm

The vertebral elements lie in a largely straight line and are mostly complete but variably preserved. The atlas, axis and first cervical vertebra are in articulation with each other and the skull and are removed together as single block (NMS G.2021.6.2). The other cervical vertebrae are visible in dorsal view on the main slab NMS G.2021.6.1 (Figs. 1A, 6A). The bulk of the dorsal vertebrae are exposed in the ventral view on the main counterslab, NMS G.2021.6.3 (Fig. 6C). Caudal vertebral elements and impressions are split between the slab and counterslab, NMS G.2021.6.1 and NMS G.2021.6.3 (Fig. 1). The cervical vertebrae are dorsoventrally flattened but lie in articulation with overlapping zygapophyses, transitioning to disarticulated dorsal vertebrae missing neural spines and rib articulations (Fig. 6A). The dorsal sections are somewhat but not severely disarticulated (Fig. 6C), likely a result of early-stage decomposition of the skeleton in a low-energy setting.

The anterior atlas-axis complex is encased in the matrix and only visible after manual μCT segmentation of NMS G.2021.6.2. The complex is composed of tubular bones with a circular opening proximal to the occipital sulcus, and it bears features that could be interpreted as dual sets of ribs the length of the complex (Fig. 6A).

NMS G.2021.6 preserves six postaxial cervical vertebrae (Fig. 6A). The vertebrae are relatively elongated for a non-pterodactyloid and vary considerably in length. The variance and irregularity in size are too considerable to be explained by preferential flattening. The third cervical vertebra is elongate, 30.4 mm in length to 16.6 mm in width (ratio of 1.8), followed by the fourth, with dimensions of 26.4 mm by 18.6 mm (1.4), the fifth 27.2 mm by 18.1 mm (1.5), the sixth 29.8 mm by 21.5 mm (1.4), the seventh 27.8 mm by 17.8 mm (1.6), and poorly preserved eighth, which forms the transition to the dorsal series on the counter slab (NMS G.2021.6.3, Fig. 6C), which is approximately 24.4 mm by 20 mm (1.22). On a continuum, the cervical series measures approximately 135 mm in total length.

There are elevated Y-shaped central regions that extend on the bodies of the cervical vertebrae, which are the only remnants of the neural processes (Fig. 6A). The degree of erosion prevents a full assessment of the diagnostic shape and height of the neural spines. The vertebrae have elongated overlapping anterolateral and posterolateral corners forming sharp prezygapophyses and postzygapophyses, bearing elevated, rugose textures likely housing elements of the transversospinalis system [58]. The shape and size of the articular facets vary in sequence, with the seventh bearing a large lunate anterior articulation point with the vertebral body narrowing distally with thin, bulbous postzygapophyses.

The third cervical (on NMS G.2021.6.2), which has been manually segmented from μCT data (Fig. 6B), can be described in detail. It preserves a neural canal as a continuous oval opening and is associated with two pairs of short, thin cervical ribs. One set is ventrally placed underneath the vertebra, whereas the other stretches across the length of the cervical centrum. Surprisingly, in addition to two sets of ribs, the μCT scans revealed short, squat but distinctive post- and pre-exapophyses elevating the ventral face of the third cervical vertebra (Fig. 6B). This feature was previously associated with pterodactyloid-line pterosaurs [25] and was considered an effect of increasing body size (as noted by Andres et al. [3] when the size of the cervical vertebrae was compared with that of the sizeable non-pterodactyloid *Sericipterus*). The exapophyses have dual, asymmetrical bulbous ends that are markedly distally and anteriorly offset. The post- and preexapophyses extend 5 mm from the center of the cervical vertebra and are 8 mm wide. The presence of exapophyses in *Dearc* might reflect morphological changes associated with increased body size to provide support for the relatively heavy skull. In this case, this would be an independent acquisition of exapophyses and large body sizes relative to the same trends observed in pterodactyloids. If this is correct, then exapophyses might therefore correspond more to the size of the animal rather than being solely a phylogenetic feature, arising from convergent alterations to accommodate the increased skull size of the animal.

Nine dorsal vertebrae are well preserved (Fig. 6C, chiefly on the counterslab (NMS G.2021.6.3) in the ventral view. The anterior-most dorsal vertebra is 19 mm long anteroposteriorly, 12 mm wide mediolaterally at the widest point of the articular ends, and 6 mm mediolaterally wide at the midpoint of the centrum. The proportions reduce posteriorly: at the mid-section of the dorsal column, a vertebra is 14 mm long by 5 mm wide, with the posterior-most dorsal vertebra being just 12 mm long by 4 mm wide. The dorsal vertebrae are less deformed than the cervical vertebra, possibly due to reduced pneumatization. There is a set of morphologically distinctive, displaced vertebrae anterior to the pubic complex (Fig. 6C) on NMS G.2021.6.3. The vertebra could be unfused synsacral elements or dorsal fragments contorted by taphonomy or preferentially preserved posterior dorsal vertebra. The vertebrae in question are 14 mm long anteroposteriorly with a mediolateral width of 13 mm, considerably different and more equal in dimension than the dorsal elements anterior to them. On a continuum, the dorsal vertebra series measures approximately 235 mm in total length.

In NMS G.2021.6.1, the caudal vertebrae of the tail are poorly preserved, with the distal section of the tail covered by (and somewhat continuous with) a calcite vein. Approximately ten caudal vertebral segments can be delineated, resulting in a total measurable tail length of approximately 190 mm. The two anterior caudal vertebrae can be most clearly distinguished, with preservation of one on the counter slab and its impression on the main slab. The bones are nearly equal in size and square, with length-and-width centrum dimensions of 1.1–1.4 mm. The caudal vertebra concave articulations on the distal ends for increased mobility. The anterior-most elongated caudal vertebrae of the tail are thin, with a maximum mediolateral width of approximately 3.4 mm and slightly flared articulation points.

### Ribs

The vertebral elements are surrounded by disarticulated curving dorsal ribs spread left and right of the body, dispersed on both main slab and counterslab NMS G.2021.6.1 and NMS G.2021.6.1.3 (Fig. 6C). The cervical ribs revealed by the μCT scan of the third cervical vertebra are described above. The best-preserved visible ribs lie on the counterslab NMS G.2021.6.3. The best-preserved anterior dorsal rib is 10 mm wide laterally at its head and 4 mm proximally, reducing to less than 2 mm distally. The ribs are variably semi concave (160°), and none preserve their full length. There are approximately 20 thin bones preserved as either impressions or fossil bone spread across the slab. These rib elements lie in proximity but are disarticulated and contorted without preferential direction. The ribs are morphologically simple, have slight concavity or a medial line, and vary in maximum width from 2 mm to less than 1 mm. The variance might be due to the bones being ribs or gastralia. Ornamental, leaf-like, sternal ribs associated with *Rhamphorhynchus* [59] are not present. For all of the ribs or potential gastralia in *Dearc*, the longest preserved element measured is a thin bone 60 mm in length.

### Scapulocoracoid

The shoulder girdle spreads across the two main slab and counterslab, NMS G.2021.6.1 and 3 (Fig. 1A), with the elements lodging in the matrix at an angle preventing good visual access and measurements of full dimensions. The proximal end of the right scapula and distal end of the left coracoid are preserved in dorsal view on the main slab (NMS G.2021.6.1), whereas a ventrally preserved, poorly visible articulation point between the two is observed on the counterslab (NMS G.2021.6.3). The partial sections of both bones are straight. The left coracoid measures 40 mm proximodistally with a width of 5.5 mm at the medial point. It has a ventral elongate furrow spanning distally from the glenoid. The counter slab shows complete ossification in the scapulocoracoid complex at the glenoid, which is interpreted as an indicator of maturity [28]. The right coracoid is slightly better preserved, with a total preserved length of 51.6 mm, flaring distally from 5 to 11 mm, and tapering with distance. The distal/sternal expansion of the coracoid is common in most pterosaurs, with rare exceptions (i.e. *Cratonopterus* [60]). It is seen in taxa ranging from an early branching *Dimorphodon* (NHMUK R.1034 [23]) to later-branching *Bellubrunnus* (BSP XVIII–VFKO–A12 [61]), *Rhamphorhynchus etchesi* (MJML K-1597 [62]) and *Sericipterus* (IVPP V14725 [3]), there is a marked sternal expansion in monofenestratans such as *Darwinopterus* (i.e., HGM 41HIII-0309A) and especially *Ceoptera* (NHMUK PV R37110) [6]. Given that the distal end is not exposed well and is covered by the sternum, the diagnostic morphologies of the sternal articulation facets or flanges cannot be interpreted. The exposed lateral body shows a triangulate morphology rather than the tubular shape associated with monofenestratans [5]. The ventral preservation orientation also prevents assessment of the *m. biceps* origin or the presence of a ventral tuberosity on the coracoid as an origin point for *m. coracobrachialis* [3, 23, 63].

### Sternum

The main slab (NMS G.2021.6.1) contains a sternum. It is a thin (not thicker than 2 mm), relatively featureless, broad plate-like bone that lodges between the scapulocoracoid complex. This bone usually ossifies late in pterosaurs [64] and tends to be poorly preserved and, at times, completely absent. In NMS G.2021.6.1, the sternum is wider (34 mm) than long (23 mm) and probably has yet to completely ossify posteriorly [64]. For a sizeable animal with a large requirement of musculature support, the sternum of *Dearc* is small (3% of the total skeletal length). The sternal plate has a smooth dorsal surface, without marked ridges or topography. No cristospine is preserved, but there is a dual, symmetric anteriorly prominent tubercle, which could have functioned as a coracoid articulation. Something unusual in *Dearc* is that the sternal plate extends anteriorly, forming an overall heart-shaped morphology. Given the often poor preservation and observability of pterosaur sterna, it is difficult to make solid comparative assessments with other pterosaurs, but we here consider this to be an autapomorphy. The sternum in *Dearc* clearly differs from the triangulate shape described by Padian [29, 64] in *Dorygnathus*. Its anterior margin is dissimilar to the horizonal edge seen in adult *Rhamphorhynchus* or the heavily incised condition in subadults and juveniles [27]. The anteriorly deflected anterior edges of the plate, however, can be seen, albeit to not to as an accentuated degree in non-pterodactyloids such as *Scaphognathus* (SIPB Goldfuß 1304a) and *Nesodactylus* [65]. Arguably, the same morphology can also be found in pterodactyloids, such as *Pterodactylus* or *Ardeadactylus* (as outlined by Hone, 2023 [64]). These comparisons with *Dearc*, therefore, hint at the morphological diversity of pterosaur sterna, even in closely related and postcranially morphologically conservative clades.

### Humerus

**Fig. 7:**
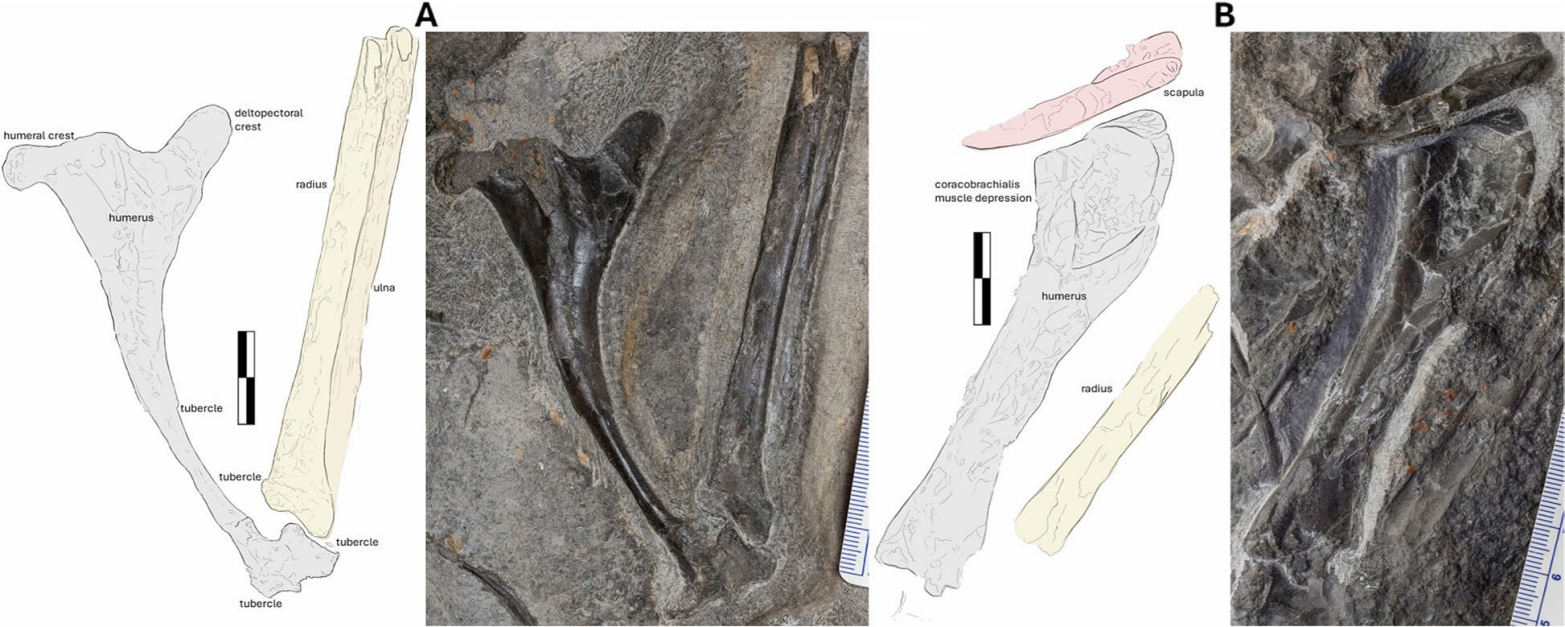
The humerus of NMS G.2021.6. The photograph of paired humerus, ulna and radius, with an interpretative annotated illustration and photograph. **A** the right humerus NMS G.2021.6.1and the left, **B** NMS G.2021.6.3. Scale bar 20 mm

Both humeri are preserved, with the right exposed on the dorsal side of the main slab (NMS G.2021.6.1) (Fig. 7A) and the left preserved in a ventral orientation on the counterslab (NMS G.2021.6.3) (Fig. 7B). The proximodistal length of the right humerus is 112 mm, with a slender diaphysis of varying width from 35 to 20 mm at the proximal and distal ends, respectively, measuring just 9 mm medially. The slender diaphysis curves strongly by ~ 150°, with a smooth shaft and delineated putative muscle attachments. The deltopectoral crest of the right humerus is a simple proximally extending lobe diverging from the diaphysis with a shallow angle of 134°. The deltopectoral crest is higher than the margin of the ulnar crest (aka. humeral head), it is lobate with a rounded proximal margin. It separates from the humeral head via a shallow U-shaped sulcus. The ulnar crest of the right humerus is bulkier than the deltopectoral crest. Its apex is flat, laterally transforming into a bulbous extremity. The individual condyles of the distal right humerus are hard to delineate and are only visible in general morphology due to poor preservation in the dorsoanterior view (Fig. 7A). The distal articular end has three lunate surfaces, one in direct articulation with the ulnar proximal epiphysis, the other ventrally oriented and the last in line with the medial crest.

**Fig. 8:**
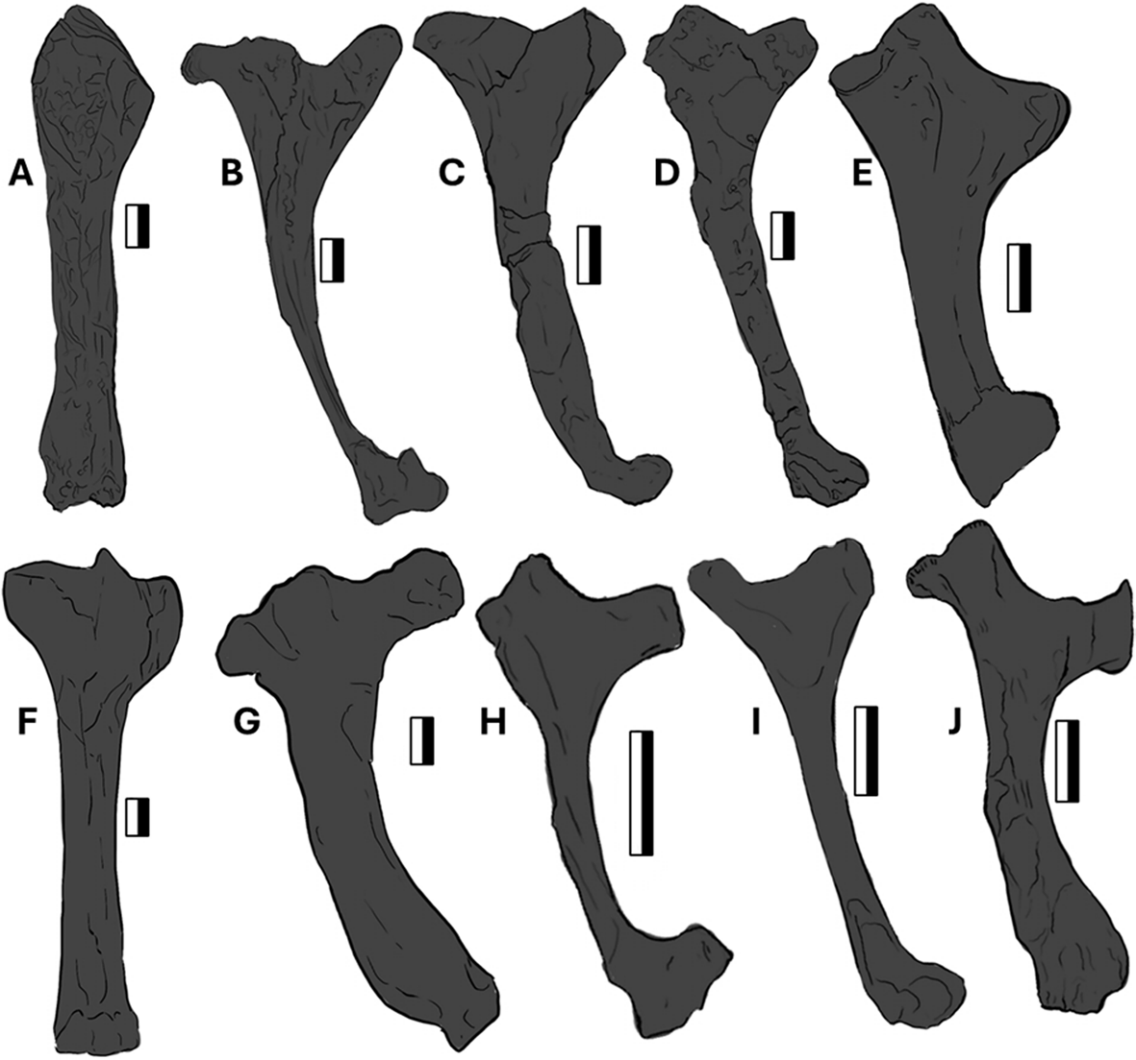
Comparative pterosaur humeral morphologies. From left to right, outlines of pterosaur humeri. **A** *Dearc* (NMS G.2021.6.3, left ventral); **B** *Dearc* (NMS G.2021.6.1, right dorsal); **C** Scaphognathinae indet. (OUMNH J.23043); **D** *Sericipterus* (IVPP V14725); **E** *Rhamphorhynchus muensteri* (NHMUK PV OR37002); **F** a sizeable pterodactyloid (SMNK PAL 6990); **G** Rhamphorhynchidae (PRC 64); **H** *Rhamphorhynchus muensteri* (NHMUK PV R 2786); **I** *Dorygnathus* (SNHM-2911-R); **J** *Rhamphorhynchus etchesi* (MJML K-1597). 10 mm to scale

The left humerus is 118 mm long, with the width of the diaphysis measuring 15–20 mm (Fig. 7B), with a slight curvature of 170°, differing substantially from that of the right humerus. This might be due to localized flattening and subsequent deformation, as expressed by pervasive fractures, along with the fact that the two bones are exposed in different orientations. The distal ends associated with the epicondyles of the left humerus are also poorly preserved. The deltopectoral area is three-dimensionally preserved, with the crest split across two slabs. There is a shallow, oval-shaped, 30 by 15 mm depression between the deltopectoral and ulnar crests on the proximal left humerus, likely housing the insertion of the coracobrachial muscle [63, 66] (see Discussion). The sizeable, proximal extension of the deltopectoral crest over the ulnar crest is unique to *Dearc* and here is considered a newly recognized autapomorphy. Compared with those of other pterosaurs, the deltopectoral crest of *Dearc* differs from the stout, perpendicular “tongue” or hatched-shaped deltopectoral crest found in *Rhamphornonychus* [27] (Fig. 8E, H) and *Nesodactylus* [26]*.*

*Dearc* has one of the largest humeri on record in Jurassic non-pterodactyloids (Fig. 8A-B), comparable to a handful of other specimens (e.g., 90 mm—OUMNH J.23043 [25] (Fig. 8C); 100 mm—IVPP V14725 *Sericipterus* (Fig. 8D); 112 mm—PRC 64 [67] (Fig. 8G) and rivalling larger pterodactyloids 117 mm—MB.R.5591.1 [68] (Fig. 8F) or 92 mm—LF 2809 *Petrodactyle* [69]. The sizeable complete non-pterodactyloid prior to *Dearc*, *Rhamphorhynchus muensteri*, NHMUK PV OR 37002 (Fig. 8E), has a uniquely preserved three-dimensional humerus exposed in the dorsal view. The humerus size of NHMUK PV OR 37002 is 77.6 mm, catering to a 1.8 m wingspan (Hone et al. 2024 in review); it has a stockier, thicker diaphysis than *Dearc*, with a hatchet-like deltopectoral crest stemming from the humeral diaphyseal midline (as also observed in Fig. 8H, J). *Dearc* bears more similarities to other large Jurassic pterosaur humeri from the Stonesfield Slate. These include the sizeable OUMNH J.23043 (Fig. 8C), measuring 90 mm proximodistally, which is marginally shorter than that of *Dearc* but has a strongly (165°) bowing diaphysis, albeit with more robust and simpler crests. This bone (OUMNH J.23043) was assigned to an indeterminate Scaphognathinae by O’Sullivan & Martill [25], although in comparison to the specimen described here it could belong to a *Dearc*-grade animal. The *Dearc* humerus bears similarities to the sizeable *Sericipterus* (IVPP V 14725, Fig. 8D), which also bears an accentuated diaphyseal tubercle, albeit placed more anteriorly. In *Sericipterus* the humeral crest also dominates over the deltopectoral crest. Among all pterosaur humeri, however, most similar to those of *Dearc* are select specimens of *Dorygnathus banthensis* with a proximally inclined deltopectoral crest and flat proximal ulnar crest (especially SMNS 50702 and SNHM-2911-R) (Fig. 8I), with the only difference being the reduced volume of the ulnar crest in *Dorygnathus*. The largest well-preserved *D. banthensis* on record had a wingspan of ca. 1.7 m [29] and is known from partial and poorly preserved remains, with a humeral size of 84 mm (MBR 1977.21).

### Radius and Ulna

Both the left and right radius and ulna are preserved in articulation with the distal condyle of the humerus. All are missing distal articulations and sections of the diaphysis. The estimated total length of the bone measures 90.8 mm for the right ulna and 113 and 120 mm for the right and left radius (Fig. 7A), respectively. Both the ulna and the radius are straight and do not undulate in thickness across the diaphysis, with the radius gently flaring ventrally at the proximal articulation. The proximal articulation of the right and left radius protrudes further than the ulna; its ventral articular end extends laterally and is bulbous with a rugose texture. This trait has not been observed in other non-pterodactyloids, save for a particularly distinctive *Rhamphorhynchus*, *R. etchesi*, MJML K-1597, with “an enlarged process extending away from the diaphysis, giving it a slight L-shaped appearance” [62]. A “boot-shaped outline” has been noted in other pterosaurs, including *Rhamphorhynchus* [62] and *Dorygnathus* [29]. This feature has been reduced in size to a notch in *Sericipterus* (IVPP V14725) [3] or described as a “simple, rounded, slightly dished facet” in the darwinopteran *Ceoptera* (NHMUK PV R37110 [6]) and remains unremarkable in Jurassic pterodactyloids (e.g. *Pterodactylus antiquus*, DMA-JP-2014/004).

### Metacarpals

**Fig. 9:**
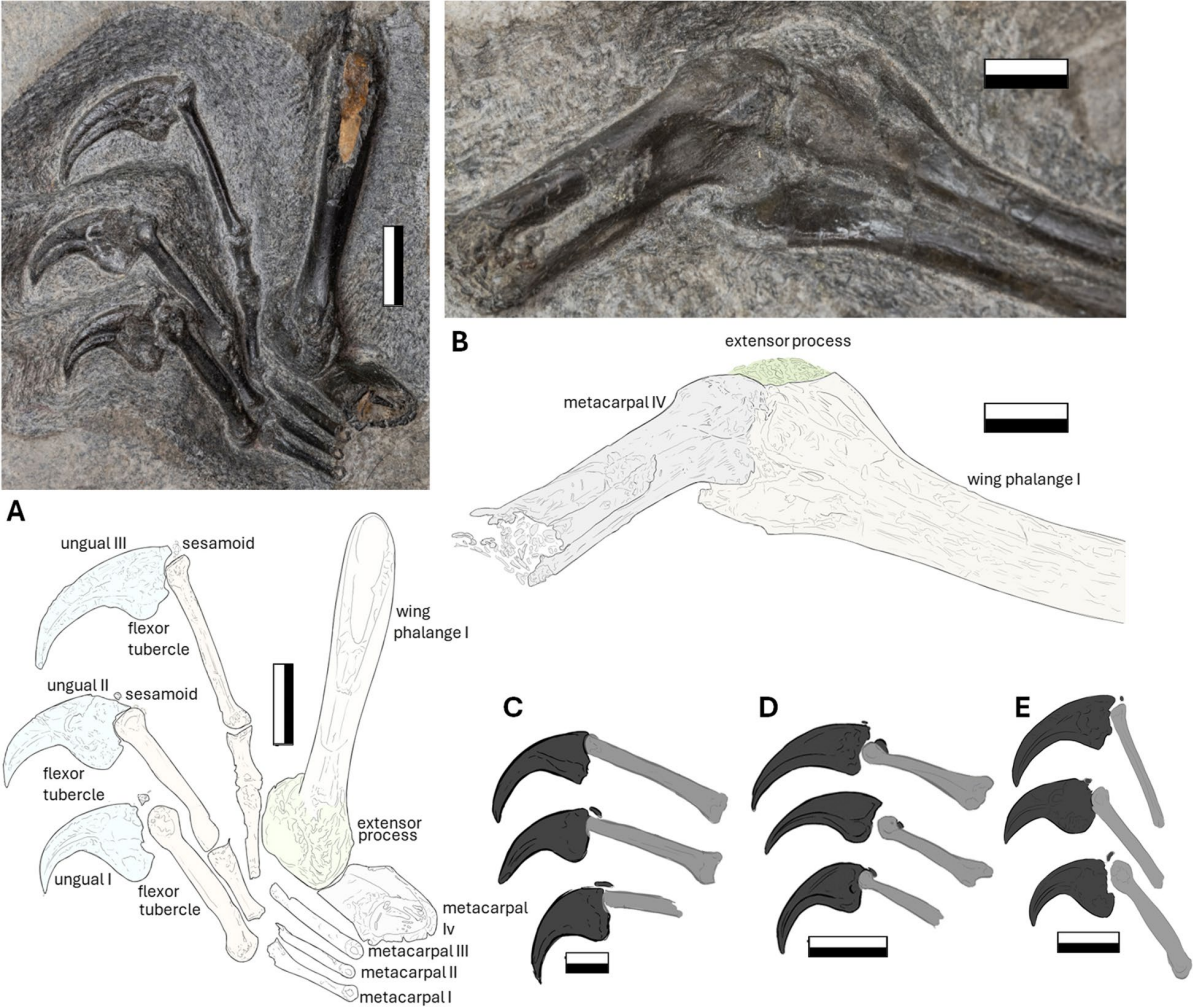
The manual apparatus of NMS G.2021.6.1. The right, **A** and left, **B** interpretative illustration and photographs of the metacarpal and manual region of NMS G.2021.6.1. The line art of manual unguals of: **C** *Dorygnathus* (MBR 1920.16); **D** *Darwinopterus* (IVPP V16049); **E** *Dearc* (NMS G.2021.6.1). Scale bar 10 mm

Portions of the metacarpals are preserved for both the left and right manus on the main slab NMS G.2021.6.1, although no complete metacarpals are present. The right set is best preserved of the two, with the left being severely eroded, preserving only a large portion of the fourth metacarpal. The right metacarpals set are tightly bound, and metacarpals I-III are thin and equal in size (Fig. 9A). The preserved portions of the right metacarpals measure no less than 11 mm in proximodistal length, with distal apices obscured and the proximal ends eroded away. The medially eroded metacarpals I-III reveal 0.5 mm thick bone walls with small round pneumatic cavities.

The fourth, more robust, metacarpals are also preserved in differing orientations on NMS G.2021.6.1 (Fig. 9A-B). The left metacarpal lacks proximal articulation, measuring approximately 38 mm proximodistally (Fig. 9B). Its mediolateral width varies from 9 mm proximally to 8 mm medially and 14 mm wide distally, with a 7 mm dorsoventral depth. The distal articulation of the left metacarpal has a robust bicondylar ginglymus, with a hooked laterally flat dorsal condyle (Fig. 9B). The less prominent ventral condyle is separated by a shallow sulcus extending down the body, which houses a pneumatic foramen and anchors tendons for wing mobility. The right metacarpal IV largely obscures the wing phalanx in articulation and is visible from a relatively uninformative proximal perspective (Fig. 9A). Given the in-articulation alignment of the ulna and radius to the metacarpals, the approximate total length of the fourth metacarpal can be estimated to be between 40–75 mm, making it 30–60% of the humerus length. It is therefore unlikely to be of pterodactyloid grade, as pterodactyloids have a fourth metacarpal 80% or more of the humeral length [22]. The borders of the metacarpal shaft appear to be without marked rugose protuberances and are not strongly undulating in thickness (8–9 mm) across its preserved length. The same condition, of a long straight shaft with large distal condyles, can be seen in *Rhamphornychus* [62], *Bellubrunnus* [61] and *Dorygnathus* (NHMUK PV R 10087/5313; MBR 1920.16) [29], although in *Dearc,* there is no sign of the crista metacarpi, as observed in *R. etchesi* (MJML K-1597). The distal condyles in Early Jurassic pterosaurs, such as *Dimorphodon* (NHMUK R.1035) and *Campylognathoides* (SMNS 54049), while also asymmetrical, are narrower and less pronounced, with the proximal end being more robust in comparison to the distal.

### Manus

The right manus is exceptionally preserved and retains its three-dimensional topography on NMS G.2021.6.1. Digits I-III are completely preserved, and a portion of the wing finger (digit IV) is also present. The individual digits increase in length from digit I (24 mm), II (33 mm) to III (41 mm) (Fig. 9A). The manual unguals have visible concave flexor tubercles, semilunate triangulate depressions of the extensors and shallowly hooked proximoventral heels (Fig. 9A). There are two bony sesamoids located above the ungual anterior crests of the digits. These sesamoids have been observed across early and late groups of non-pterodactyloid pterosaurs, from *Eudimorphodon*, *Dimorphodon*, and *Dorygnathus* [70] to *Darwinopterus* and *Kunpengopterus* [71]. All unguals have deep lateral grooves originating from the mid-section of the bone. The first ungual is the largest, measuring 22 mm proximodistally from the extensor lip to the tip. It is 9 mm tall dorsoventrally at the highest point (from the flexor tubercle to the outer curvature); ungual two is 16 mm and 9.5 mm tall; and ungual three is 15 mm and 10 mm tall. The unguals become taller but shorter from I to III, the third being the longest of the hand, retaining an accentuated curvature, with the first being short and stocky (Fig. 9E). The morphology is similar, along with the inner and outer ungual curvature (explored later in the discussion), to the manual unguals ranging from the early branching *Dorygnathus* (i.e. MBR 1920.16, Fig. 9C) and late branching *Darwinopterus* (i.e. IVPP V16049, Fig. 9D) specimens).

### Wing phalanges

**Fig. 10:**
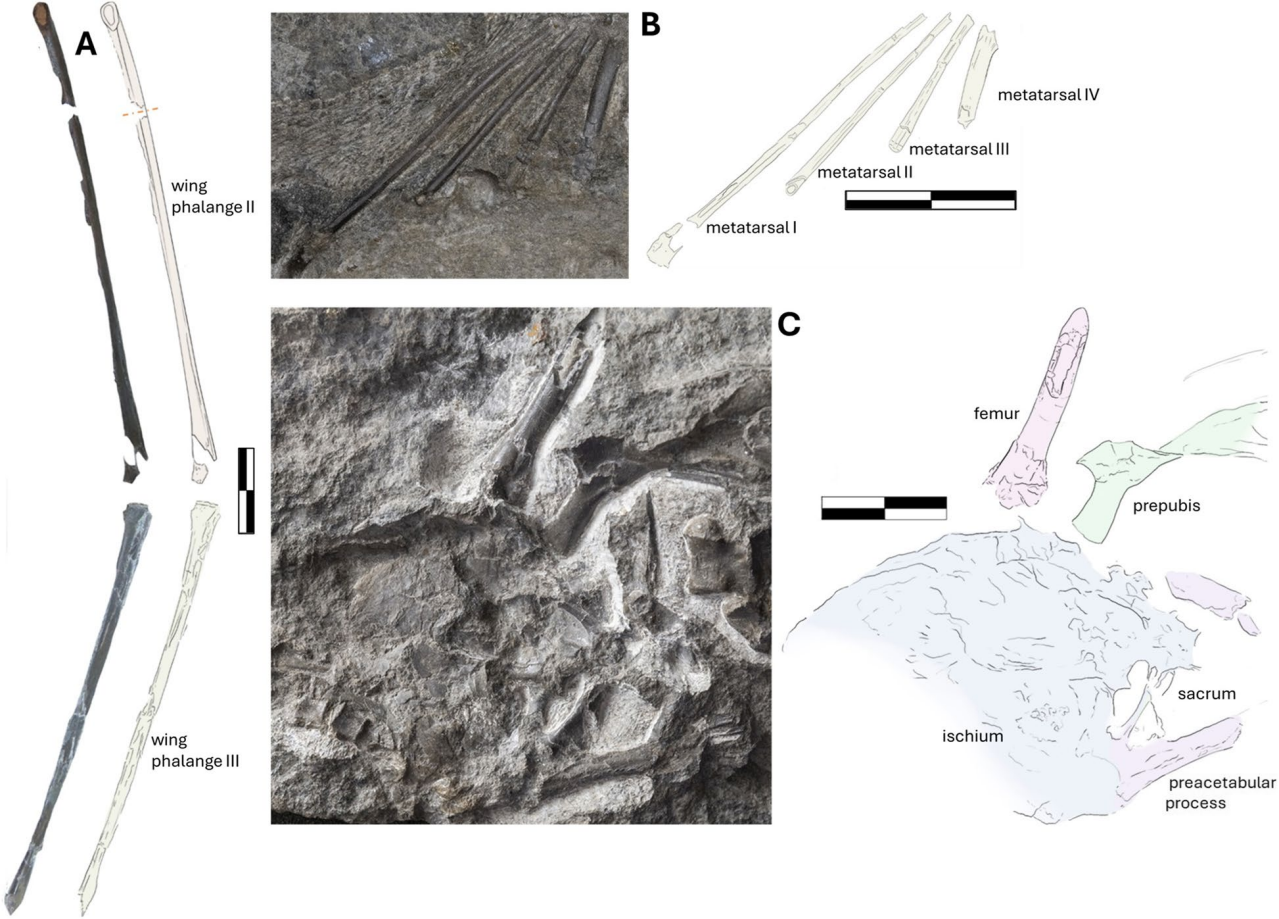
The postcranial elements of NMS G.2021.6. The photograph and illustration of the wing phalange, **A** two (NMS G.2021.6.3) and three (NMS G.2021.6.4). The photograph and illustration of the pedal metatarsal, **B** NMS G.2021.6.1. The ventral surface of the pelvis, **C**, NMS G.2021.6.3, with photograph and interpretative illustration. Scale bar 20 mm

The wing phalanges are all partially preserved, each missing at least one of their terminal ends, and do not provide a full-length measurement for diagnostic assessment. The first left wing phalanx is preserved in posterolateral view on the main slab (NMS G.2021.6.1). The bone has a large rugose extensor process and a lobate lip-like flexor process (Fig. 9B). Both create topographic heights that open to a medial groove extending from the intercondylar sulcus running down the preserved length of the first phalanx (Fig. 9B). A similar shallow posterior groove is also observed in another Scottish pterosaur (NHMUK PV R1362, from the Late Jurassic of Eathie) [72] or *Nesodactylus* [65], this character was considered to be a rhamphorhynchine-diagnostic feature [25, 72]. The phalanx has a preserved length of 109 mm and is crushed and expanded distally. The right first phalanx is preserved in a front-facing proximal view (also on NMS G.2021.6.1), with a rugose multilobate extensor process, which has been truncated by tidal erosion, exposing a perpendicular cross-section with thick bone walls (4 mm at the thickest point compared with the 13 mm radius). The extensor processes on both phalanges are ossified to the shaft, a sign of maturity that may seem inconsistent with the young histological age of the specimen [1] (Fig. 9A-B), with the extensor insertion site having a mottled and rugose texture different from the body of the phalanx. The second right phalanx is visible in the lateral view on the side of NMS G.2021.6.3. Its total preserved length is 135 mm. The bone gradually becomes thinner (12 mm to 10 mm) distally, and it terminates with a poorly preserved articulation point (Fig. 10A). The articulation area is flat. The third right wing phalanx is also preserved in the lateral view and articulates with the preceding phalanx. It is the only bone preserved on the NMS G.2021.6.4 slab. It is poorly preserved and fractured. It looks slightly curved, but this might be due to fracturing. The preserved segment is 129 mm in length, becoming thinner distally from over 10 mm to 9 mm. The fourth wing phalanx is absent on both wings.

### Pelvis

The pelvis is preserved in a relatively uninformative ventral orientation on the counterslab, NMS G.2021.6.3 (Fig. 10C), limiting the scope of comparative and anatomical assessment. UV photography was used to aid delineation of the bone margin but lack of fluorescence made the attempt unsuccessful. The elements overlap or vanish into the matrix, with only the partial margins of the paired preacetabular processes of the ilium being clearly observable (Fig. 10C). The left preacetabular process is preserved in a better condition than the right. The process is 32 mm long and 5 mm wide with a slender shaft and a lobate proximal end. The ischium is a conical amorphous plate with a barely visible margin. The preserved ischium is approximately 50 mm long its sacral plate comprises approximately 25 mm its total length. There is no sign of sacral vertebral articulations, save for the displaced fused set of vertebrae anterior to the pubic complex. NMS G.2021.6 retains a sacral plate with two sets of paired oval fenestrations. This element is crushed and hard to delineate, its morphological details are unclear.

### Prepubes

There is a connected pair of hatchet-shaped prepubes near the sacrum of the counterslab NMS G.2021.6.3 (Fig. 10C). Two prepubic bones connect at their proximal points, with the left better preserved than the right. The best preserved is 25 mm long in maximum dimension with a width of 4 mm in the midsection and 16 mm at the proximal end. It flares distally. The proximal end is eroded, preventing a full comparative assessment, but differs from the slender, tabular morphology associated with *R. muensteri* (MB-R. 3633.1–2 [59]). The proximal heads look more like those of *Dorygnathus* (i.e., SMNS 50164) [29] and *Scaphognathus* (SMNS 59395)*,* with shallow asymmetrical extensions, and unlike those of pterodactyloids such as *Germanodactylus* [73], whose proximal articulation points are larger and lobate. The prepubic pair anchors 1 mm long, thin, interweaving bones, which likely compose part of the gastral basket. If the slender bones are gastralia, the bone morphology would differ substantially from the ornamental leaf-like morphology gastralia of *R. muensteri* (MBR. 3633.1–2) [59].

### Femur

The proximal end of the femur is preserved and overlapped by the pubic plate on the ventral counter slab (NMS G.2021.6.3) (Fig. 10C). Only 30 mm of the bone is preserved, with a 4 mm wide shaft and 1 mm thick bone walls. Little can be said about its morphology or its similarities and differences with other pterosaurs.

### Metatarsals

The left metatarsals are present on NMS G.2021.6.1, the main slab, and are not enclosed in a tight bundle but splay distally (Fig. 10B). No proximal articulation is visible, with the mid-section of the metatarsals being the only aspect observable. The fourth metatarsal is considerably thicker and more robust than the preceding three (1.2–1.6 mm in thickness to almost 3 mm in the terminal metatarsal). This is considered an autapomorphy of *Dearc* [1]. In *Dorygnathus*, apart from the proximal articulating ends, the bodies of metatarsals I-IV are slender, with the first appearing slightly more robust (SMNS 51827), with the same conditions as those in *Rhamphornychus* (Abb. 17 in Wellnhofer, 1975 [27]) and remained consistent in other non-pterodactyloids [71].

## Discussion

The pterosaur fossil record is marred with enormous temporospatial gaps, barring our understanding of wider evolutionary and morphological trends. However, even partial specimens from poorly sampled intervals can have an enormous impact on our understanding of macroevolutionary events. *Dearc* *sgiathanach* (NMS G.2021.6) of the Lealt Shale Formation shines light on a ‘dark age’ of pterosaur evolution—the Middle Jurassic. NMS G.2021.6 is an important specimen for understanding pterosaurs, given that it is largely complete, articulated and preserved in three dimensions, exceedingly rare for large Jurassic pterosaurs. Its discovery solidified a previously proposed idea that the Jurassic pterosaurs attained broader wingspans (over two meters) and larger body sizes as early as the Middle Jurassic [1, 25]. The vital temporospatial placement and exceptional preservation of NMS G.2021.6.1 necessitate a detailed osteological study of both the cranium and antebrachium.

### Cranial musculature

While there is a substantial amount of interest in the cranial musculature of dinosaurs (e.g., [8, 15, 74, 75]), pterosaurs have been less studied. A handful of important papers have been published over the past two decades, but they are relatively few in number (e.g., [14, 53, 57, 77]). The most exhaustive paper exploring this topic is a recent study of osteological correlates and bite force estimation in Cretaceous pterodactyloids [14]. Given the three-dimensional preservation of the *Dearc* holotype, the principal cranial muscle attachments (for adductors responsible for jaw closure and depressors for jaw opening) can be inferred.

**Fig. 11:**
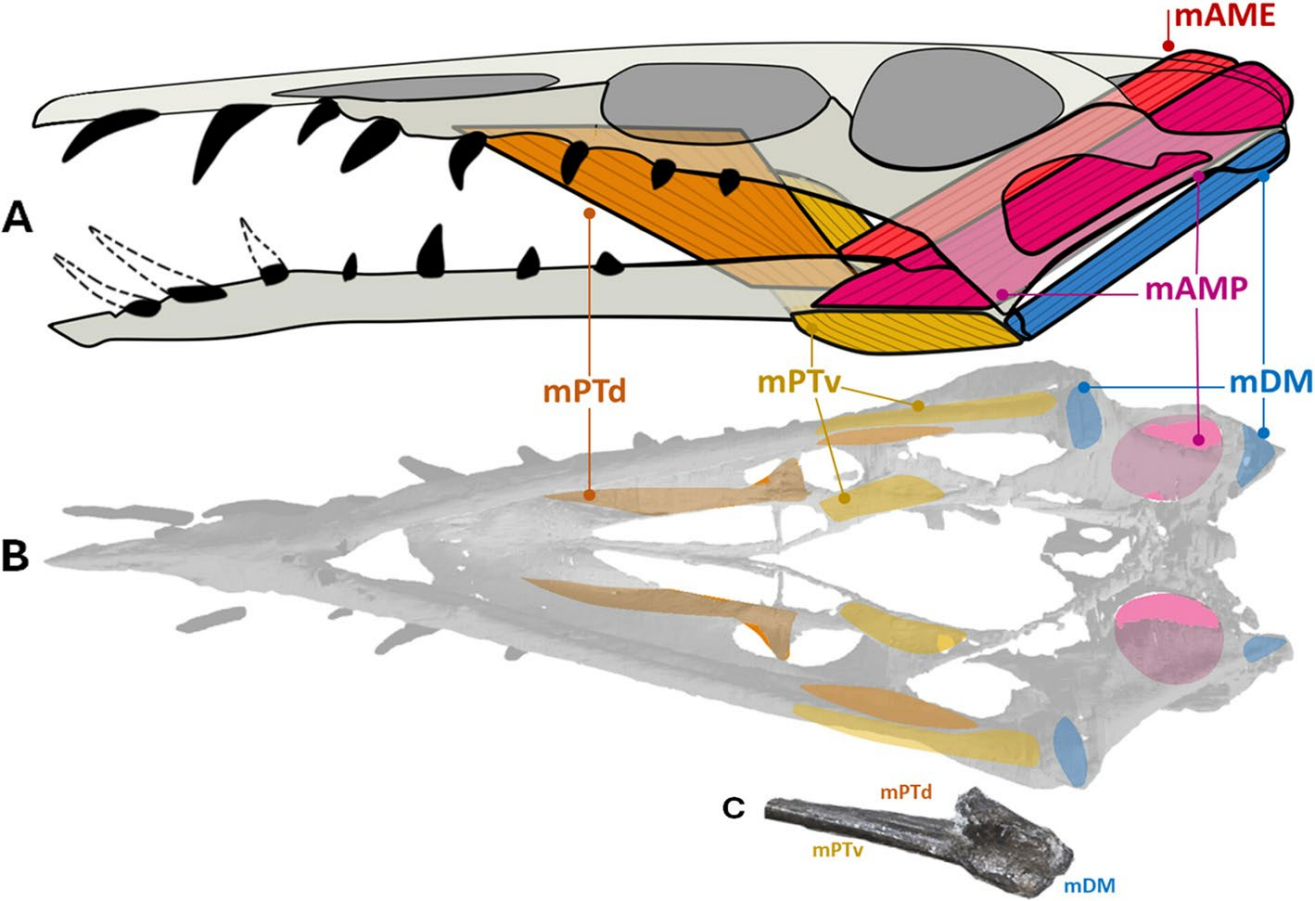
The reconstructed cranial musculature of NMS G.2021.6. The simplified reconstructed skull musculature, **A** with the origin and insertion location of muscles highlighted, **B**. The photograph of ventral distal dentary, **C**. Abbreviations: mAME – *M. adductor mandibulae externus*; mAMP– *M. adductor mandibulae posterior*; mDM – *M. depressor mandibulae*; mPTd *– M. pterygoideus dorsalis*; mPTv – *M. pterygoideus ventralis*

The fine preservation of the palate in *Dearc* permits mapping of the internal palatal adductors (*M. adductor mandibulae internus*) using Extant Phylogenetic Bracketing (EPB). The muscle origin and insertions are inferred using animals set across the archosaurian extant phylogenetic bracket (as developed by Witmer & Thomason [9]). The interpreted origin of *M. pterygoideus dorsalis* (mPTd) is clearly visible. In crocodilians and birds [11, 15], mPTd originates from the dorsal pterygoid, palatine, ectopterygoid and tendinous jugal attachments [77]. In *Dearc*, the area associated with the origin of the muscle is marked with a sizeable ridge-flanked, depressed, smooth surface anterior to the choana (Fig. 11B). Caution must be taken, however, as similar “smooth and excavated surfaces” can be related to air sinuses and passages [11]. *M. pterygoideus dorsalis* has been described as leaving “well-defined muscle scars” in pterosaurs [14]. The area associated with the insertion of mPTd is anteriorly located in the antorbital midsection and might explain the peculiar ectopterygoid shape. The muscle likely traverses one of the enlarged palatine fenestrae to insert on the medial surface of the angular, located on the mandibular midline in most non-pterodactyloid pterosaurs [11, 76].

The mandibular extensor muscles originate from the bones surrounding the upper temporal fenestra in archosaurs but do not always leave obvious osteological correlates [14]. The temporal adductors are set almost perpendicular to the long axis of the dentary, usually parallel to the quadrate [57]. In the *Dearc* holotype, the *m. adductor mandibulae externus* (mAME) angle was approximately 35–50° (measured from the horizontal plane posteriorly), which is close to that observed in *Rhamphorhynchus* (40°) and *Angustinaripterus* (55°) [57]. The mAME muscles were supported by a frontoparietal crest in more derived pterosaurs [14, 57]. The region hosting this crest (apex of the skull) might have been eroded away in the *Dearc* holotype, but the crest is generally absent in Jurassic non-pterodactyloid pterosaurs, so we can infer that it was most likely absent in *Dearc*. The retroarticular process of the jaw is also reduced (Fig. 11C) and not as well defined as it is in Triassic durophagous pterosaurs (e.g., *Raeticodactylus* & *Caviramus* [54]). The palatal musculature must have been well developed in *Dearc*, given the large muscle attachment area, the muscles could possibly compensate for the relatively reduced power of the external temporal adductor muscles (such as the mAME) (Fig. 11B).

Most pterosaurs were incapable of complex kinesis within the skull, unlike the highly mobile skulls and beaks of modern birds [78]. This also applies to *Dearc*, given that it has a fully ossified cranium in which individual bones are tightly sutured together, save for joints on the basipterygoid processes articulating with the pterygoids, as also seen in other non-pterodactyloid pterosaurs [78].

### Brachial-antebrachial musculature

The *Dearc* holotype preserves almost the full set of brachial-antebrachial bones in differing orientations. Even though the individual bones are not always complete, they are mostly finely preserved in three dimensions. This allows for the identification of osteological correlates of soft tissues, particularly musculature, inferred via the archosaurian EPB as described in the previous section (as developed by Witmer & Thomason [9], for our antebrachial investigation using crocodilians [79], birds [80–82], and inferences made on extinct animals [16]. Some osteological correlates are especially markedly defined and morphologically unique.

The humeral peculiarities in *Dearc* include a large humeral and deltopectoral head, set on a slender elongate humeral body with topographic delineations. The expansive, lobate deltopectoral crest anchored principal flight musculature, such as *M. pectoralis* (mP)*.* The pectoral muscle acted as an anchor for a broad superficial extrinsic muscle that covered much of the ventral torso of the animal [16, 79, 83]. In crocodiles, the muscle is responsible for maintaining limb posture [79]; in volant birds, this muscle originates from a sizeable sternal keel and inserts on the ventral surface of the proximal humerus [16, 82]. The pectoralis muscle is the largest in the avian flight apparatus, accounting for much of the muscular body mass of the animal [81] and acts as a powerful depressor for downstroke wing motion.

**Fig. 12:**
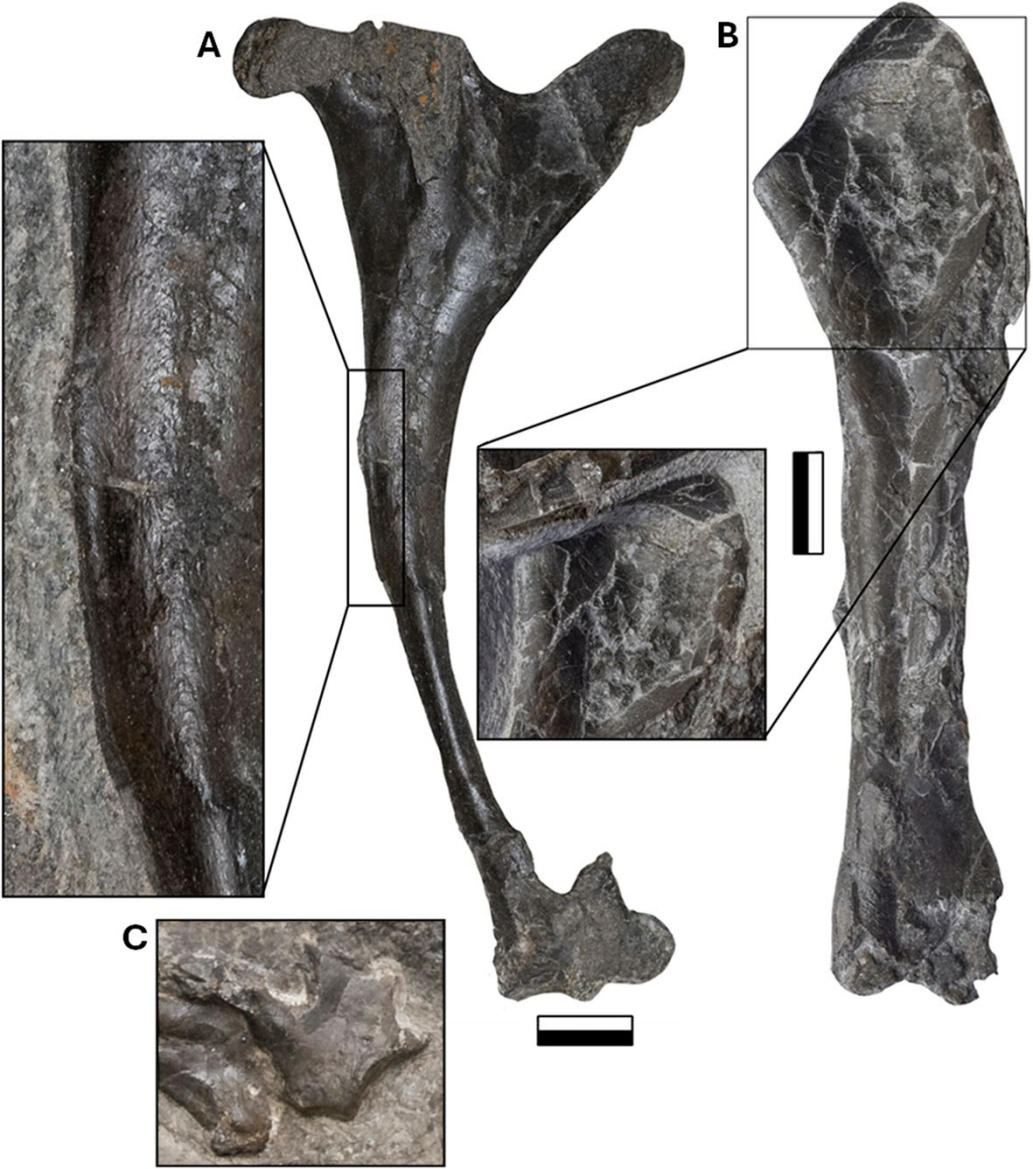
Photographs with a focus on humeral osteological correlates. The photographs of the right dorsal, **A** (laterally positioned when the animal is standing, anteriorly in flight) humerus (NMS G.2021.6.1) and left ventral, **B** (medially positioned with animal standing) humerus (NMS G.2021.6.3). The detached distal dorsal condyle of the left humerus, **C** (NMS G.2021.6.1). The boxes showcase osteological correlates

The insertion of the pectoralis at the apex of the deltopectoral crest is found across Archosauria [80], and given how sizeable pterosaurian deltopectoral crests are, this muscle was almost unequivocally present in *Dearc*. The muscle would have adducted and protracted the humerus and the shoulder. In NMS G.2021.6, the deltopectoral crest is sizeable, lobate, and curved in the anteroventral direction from the diaphysis, with some proximal deflection from the articulation region (Fig. 12A). Thus, in *Dearc,* the muscle is likely inserted and wrapped around the terminal end of the expansive crest, a position also suggested for the early branching non-pterodactyloid *Campylognathoides* CM 11424 [84], as noted in *Rhamphorhynchus* (MGUH 1891.738) (Fig. 2 in Bonde & Christiansen [85]). This attachment is consistently observed as muscle scarring on the ventral surface of the deltopectoral crest [87]. In *Dearc,* the proximally expanding nature of the deltopectoral crest morphologically differs from that observed in closely related *Rhamphorhynchus* (Fig. 8E, H). The *Rhamphorhynchus* deltopectoral crest is almost medially placed on the diaphysis and expands and flares distally [85]. This finding might suggest that various, even closely related, pterosaurs had different requirements for downstroke motion despite their comparable size and close phylogenetic affinity. The origin of the pectoralis in pterosaurs likely also stems from the sternum, and while this muscle is enormous in birds, as reflected by the sizeable sternum and keel, *Dearc* and other pterosaurs have diminutive sternal bones [64]. The sternum could have been only partially ossified in the *Dearc* holotype given the actively growing nature of the animal [1] and may have increased in size with maturity.

Another trait of note on the *Dearc* humerus is the marked osseous notch on its slender diaphysis (Fig. 12A). The region is associated with the insertion of *M. latissimus dorsi* (mLD), a superficial muscle that stretches along the vertebral column and neural spines of the posterior cervical vertebrae and anterior dorsal vertebrae in birds [16] and the dorsal vertebrae in crocodilians [79]. The muscle acts as a retractor, rotator, and extensor of the humerus [16]. Its insertion manifests across various clades as a rugose tubercle or osseous ridge on the diaphysis, as observed in crocodilians and birds [16, 79, 81, 86]. In *Anhanguera* [63] it is inferred for it to be placed on the dorsal side of the humerus posterodistal to the deltopectoral crest. Scarring attributed to this muscle has also been identified on “the posterior surface of the proximal [humeral] region” in late branching pterosaurs [87]. In *Rhamphorhynchus* (MGUH 1891.738), this muscle manifests as a long furrow [85]. In the *Dearc* holotype, this muscle attachment might manifest as an osseous notch marking the diaphyseal mid-section (Fig. 12A).

There is an oval depression on the anterior face of the partially preserved left humerus in the *Dearc* holotype (Fig. 12B), which might be associated with the coracobrachialis muscle (*M. coracobrachialis brevis,* mCB). In birds, the coracobrachial muscles (*cranialis* and *caudalis*) originate from the ventral scapula and attach to the proximal humerus [88]. The muscle acts as the wing pronator, working alongside the pectoral muscles to supinate the wing [89]. In crocodilians, the *M. coracobrachialis brevis ventralis* muscle originates from the ventral coracoid to insert on a triangular basin on the caudoventral surface of the humerus [79]. The muscle in crocodilians is multifunctional and facilitates shoulder joint flexion, humeral retraction and adduction [79]. The left humerus in NMS G.2021.6 has an elongate tear-shaped depression flanked by humeral crests on its ventral surface (Fig. 12B). Despite deformation of the element, this feature clearly has delineated topography that creates a morphological “basin.” Such a deep flange has been noted in select pterosaurs but is not universally present across the clade [84]. Interestingly, it has also been observed in large-bodied azhdarchids [66] and tapejarids [90]. The sizeable insertion scar might suggest that the muscle had to be accentuated in size and well anchored to leave such a marked impression. The osteological correlates suggest that wings, and by extension, flight patterns, might have been more reliant on adduction and protraction of the humerus in *Dearc*.

Owing to its unique humeral morphology and inferred musculature, *Dearc* may have flown somewhat differently than the similarly sized *Rhamphorhynchus*. However, we simply propose this as a hypothesis to consider in future work such as computational musculoskeletal modelling (as in [87]). With poorly preserved scapulocoracoids and metacarpals, many aspects of wing muscle morphology are not currently clear in *Dearc* and cannot be compared in detail with those of the better-studied *Rhamphorhynchus*. In addition, without numerous osteological and myological studies of similarly sized pterosaurs, it is currently difficult to untangle if the morphological alterations of some bones and muscles are phylogenetic in nature or related to increased body size and thus prone to rampant convergence.

### Manual function

In life, the ungual bones of the hand anchor keratinous claws [91], structures that perform a multitude of functions in modern animals, from digging and climbing to facilitating predation [18]. In the fossil record, preservation of keratinous sheaths is rare, unguals, on the other hand, are more likely to be preserved due to their ossified nature. The functionality of the pterosaur manus has been speculated upon since the very first pterosaur discoveries, with one of earliest reconstructions showing the non-pterodactyloid *Scaphognathus* hanging off a cliff face via its manus (Fig. 4, in Martill & Pointon [92]). Wellnhofer in his encyclopedia [40], noted the “well developed flexor processes to which strong flexor tendons were attached”, suggesting that the digits were “ideally suited for gripping and climbing steep surfaces like rocks, cliffs and tree trunks,” an observation recently corroborated by Smyth et al. [93]. Given the relative size of the unguals, the accentuated muscle correlates on the hand, and the presence of sesamoids, it is likely that pterosaurs, especially *Dearc*, used their unguals (and by extension, claws) for some function other than simply supporting the animal during terrestrial locomotion, with likelihood of facilitating arborealism, as inferred from other non-pterodactyloids [93].

Ungual shape has been studied extensively in paleontology, especially in relation to the evolution of flight or arboreality in early birds [18, 93–96] and the deduction of behavior via comparisons with modern animals. The strongly recurved shape of the *Dearc* holotype unguals falls within the observed range of perching and climbing psittaciforms, piciforms, and passeriforms [18]. In *Dearc,* the values vary from: IU (inner ungual curvature) — 114° (digit 1), 106° (2), 99° (3); OU (outer ungual curvature) – 117° (digit 1), 111° (2), 103° (3). The degree of curvature exceeds that of arboreal paravian dinosaurs closely related to birds, such as *Microraptor zhaoianus* (IU—92°; OU—90°), or early birds, such as *Confuciusornis sanctus* (IU—109°; OU—111°) (values from the third pedal digit from Cobb & Sellers [18]). Wu [97], who examined the recurved nature of pterosaur unguals via the same method, hypothesized that some pterosaurs had good grasping ability and suggested that it was useful for “arboreal, climbing, and predatory behavior”.

Large, muscularly supported and excessively curved unguals likely did not aid with flight, and because they were maintained (unlike modern birds, which lack large hand claws), they probably performed a vital function in *Dearc* and other pterosaurs. Given the well-developed sesamoids and flexor tendons, it is likely that *Dearc* was able to keep the tips of the claws elevated above the level of the substrate and maintain their functionality [70]. The fact most non-pterodactyloid pterosaurs have similar ungual morphologies (Fig. 9C-E) points to their shared functionality across clades living in different habitats and time periods. Given the similarities in these taxa, which are of varying size and phylogenetic position, this suggests scansorial or arboreal behavior in early pterosaurs [96]. As pterosaurs lack bird-style cranial kinesis [78] and highly mobile beaks, it is possible that *Dearc* and other pterosaurs employed their claws to facilitate scansorial/arboreal lifestyle and likely supplement predatory behavior.

## Conclusions

The largely complete, articulated, and three-dimensionally preserved holotype skeleton of *Dearc* provides a critical glimpse of the osteology, myology, and functional morphology of a “transitional” non-pterodactyloid pterosaur. *Dearc* shows that traits previously regarded as “derived” or “late branching” (such as the presence of exapophyses on relatively elongated cervical vertebrae) could appear in non-pterodactyloids and develop convergently, probably as a functional aid related to larger body size and modular transition. Despite the presence of “derived” traits, the specimen still retained “basal” functionalities and inferred behaviors as expressed by ungual morphology and dental wear barely differing from an Early Jurassic *Dorygnathus*. The Scottish pterosaur has a unique palatal arrangement, with a choana-intruding ectopterygoid and osteological correlates pointing to the anteriorly extending palatal musculature, which is unseen in other pterosaurs. This adaptation likely compensated for the weak bite set by reduced adductor and depressor muscle insertion areas in the posterior section of the dentary, the lack of an elevated sagittal crest and extended jaw out-lever. The palatal musculature must compensate for weak adductors, as chipped apices of teeth and enamel flaking point to a harder diet. While *Dearc* and other non-pterodactyloids pertained an overall relatively conservative bauplan, the humeral morphologies are diverse and likely affected the flight styles in non-pterodactyloid pterosaurs. Superficially, its appendicular morphology does not differ substantially from that of closely related non-pterodactyloid pterosaurs, but detailed inspection shows marked functional differences. *Dearc* had a dorsally deflected humeral head and deltopectoral crest and sported sizeable coracobrachialis and deltoid attachments. Its manual unguals were recurved and aided by the sesamoids. Despite large morphological differences, the manual unguals in non-pterodactyloid pterosaurs likely had a universal clade-wide function. The unguals could have aided scansoriality/arborealism and supplemented predatory function, although understanding their function requires more comparative studies on many animals.

NMS G.2021.6 alters the way we perceive pterosaur evolution, suggesting these flying reptiles reached notable sizes and diverse morphologies in advance of the Late Jurassic. This all points to mass diversification leading to a change in morphotypes between the Lower and Middle Jurassic. The macroscopic drivers of this change remain an open question going forward. Key specimens, like NMS G.2021.6, help to temporally constrain the region of interest. The unexpected discovery of *Dearc sgiathanach* shows that there is potential for more uniquely preserved pterosaurs to be recovered elsewhere, helping to populate geological periods and global regions previously barren, and helping to resolve the questions of macroevolutionary bauplan turnover in the Jurassic.

## Data Availability

The dataset supporting the conclusions of this article is available in the MorphoSource repository, under ID 000420007 in https://www.morphosource.org/projects/000420007 NMS G.2021.6 is accessioned by the National Museum of Scotland (Edinburgh, Scotland, UK).

## Abbreviations

BSP/BSPG: Bayerische Staatssammlung für Paläontologie, Munich, Germany
BYU: Museum of Paleontology, Brigham Young University, Provo, USA
CM: Carnegie Museum of Natural History, Pittsburgh, Pennsylvania, USA
GPIT: Institut und Museum fur Geologie und Palaeontologie, Universitat Tuebingen, Germany
GSM: British Geological Survey, Keyworth, UK
HGM: Henan Geological Museum, Zhengzhou, China
IGO: Instituto de Geología y Paleontología, Havana, Cuba
IVPP: Institute of Vertebrate Paleontology and Paleoanthropology, Beijing, China
LF: Lauer Foundation for Paleontology, Science and Education
MBR: Museum für Naturkunde, Humboldt, Germany
MGUH: Geological Museum, Copenhagen, Denmark
MJML: Museum of Jurassic Marine Life, Kimmeridge, UK
MTM: Hungarian Natural History Museum, Budapest, Hungary
NHMUK: Natural History Museum London, UK
NMS: National Museums Scotland, Edinburgh, UK
OUMNH: Oxford University Museum of Natural History, Oxford, UK
PRC: Palaeontological Research and Education Centre, Mahasarakham University, Maha Sarakhaml, Thailand
SMNK: Staatliches Museum für Naturkunde Karlsruhe, Karlsruhe, Germany
SMNS: Staatliches Museum für Naturkunde Stuttgart, Stuttgart, Germany
SNHM: State Museum of Natural History in Brunswick, Saxony, Germany
TMM: University of Texas Vertebrate Paleontology Collections, Texas, USA
WDC: Wyoming Dinosaur Center, Thermopolis, USA
ZDM: Zigong Dinosaur Museum, Sichuan, China
